# Effectiveness of Indapamide Prolonged‐Release and Perindopril Versus Perindopril Monotherapy for Treated Uncontrolled Hypertension: A Target Trial Emulation

**DOI:** 10.1002/pds.70295

**Published:** 2026-02-08

**Authors:** Céline Darricarrere, Virginie Simon, Manel Pladevall‐Vila, Emmanuelle Jacquot, Morgane Ballon, Marie Mangin, Dominique Procureur, Jaume Aguado, Xabier Garcia‐Albeniz

**Affiliations:** ^1^ Servier Suresnes France; ^2^ RTI Health Solutions Barcelona Spain; ^3^ The Center for Health Policy and Health Services Research, Henry Ford Health System Detroit Michigan USA; ^4^ IT&M Stats Neuilly‐sur‐Seine France; ^5^ R&D Life Cycle Management Suresnes France

**Keywords:** antihypertensive treatment effectiveness, combination therapy, real‐world evidence, target trial emulation

## Abstract

**Objective:**

To assess the effectiveness of indapamide prolonged release and perindopril in combination using blood pressure (BP) records collected in routine practice.

**Methods:**

Using a target trial emulation framework, an observational retrospective cohort study was conducted. The data source was the United Kingdom's CPRD Aurum general practice database. Adults with systolic BP (SBP) ≥ 145 mmHg treated with perindopril 4/5 mg for ≥ 4 weeks at a stable dose who either added indapamide 1.5 mg to perindopril (*n* = 193) or continued on perindopril monotherapy (*n* = 14 571) were included. Balance between treatment arms was achieved with propensity score matching; results were explored in additional analyses using different eligibility criteria and alternative statistical methodologies. The primary outcome was change in SBP from baseline to Week 8 between indapamide added to perindopril versus perindopril monotherapy.

**Results:**

In the primary analysis, indapamide added to perindopril yielded an additional SBP reduction of −6.3 mmHg (95% confidence interval [CI] −8.7 to −3.9) over perindopril monotherapy at Week 8. Results of additional analyses were consistent with the main analysis, but effect estimates varied due to diverse underlying assumptions.

**Conclusions:**

Target trial emulation allowed assessment of antihypertensive treatment effectiveness, and indapamide plus perindopril yielded clinically meaningful decreases in SBP over perindopril monotherapy. Secondary and sensitivity analyses support that these findings were robust.

## Key Points

- Adults with a systolic blood pressure ≥ 145 mmHg after at least 4 weeks of treatment with perindopril 4/5 mg per day who added treatment with indapamide 1.5 mg presented a reduction in systolic blood pressure of −6.3 mmHg on average (95% confidence interval, −8.7 to −3.9) over perindopril monotherapy after 8 weeks of treatment.
- Sensitivity analyses that challenged the assumptions made to implement this analysis in the United Kingdom's CPRD Aurum general practice database yielded consistent results.

## Plain Summary Language

Treatment of increased blood pressure (hypertension) relies primarily on oral antihypertensive medication. When hypertension it is not well controlled with a single drug, medications with different mode of action can be combined. In this study data coming from routinely collected medical information in the UK was used to study the effect of adding indapamide (1.5 mg) to perindopril (4/5 mg) in patients whose hypertension was not well controlled with perindopril alone. The results are highly compatible with a decrease in blood pressure from −8.7 to −3.9 mmHg after 8 weeks of treatment.

## 1 Introduction

Hypertension is a major cardiovascular risk factor, and reduction of blood pressure (BP) can decrease mortality and mortality due to cardiovascular disease [1]. In the United Kingdom (UK), the prevalence of hypertension was 30% in men and 26% in women in 2018 [2]. A primary reason for uncontrolled hypertension is that many patients receive only monotherapy [3, 4], although patients may benefit from multiple therapies to achieve optimal and sustained BP control [5]. Based on NICE guidelines, treatment should be initiated with an angiotensin‐converting enzyme inhibitor, angiotensin receptor blocker, or calcium channel blocker, depending on patient profile. If BP control is inadequate on a single agent, a second drug should be added, such as a thiazide‐like diuretic or a calcium channel blocker, if not already used in the first line [6]. For example, the combination of the antihypertensive treatments perindopril (an angiotensin‐converting enzyme inhibitor) and indapamide (a diuretic drug) may yield better control of hypertension compared with monotherapy [7, 8, 9].

Antihypertensive treatment efficacy is typically evaluated in randomized controlled trials (RCTs) with protocolized, iterative measuring of BP. When RCTs are not feasible or pragmatic, observational analyses using real‐world data (RWD) can provide evidence on the comparative effectiveness of potential interventions [10, 11]. With explicit specification of the hypothetical target trial and rigorous statistical methods to control for biases and confounding, observational analyses can elicit similar estimates of treatment effects as would be achieved with an RCT [10]. Within a target trial emulation framework, BP measurements from electronic medical records (EMRs) are a valuable data source for assessing the antihypertensive effectiveness of treatments used in routine care.

The objective of this study was to estimate change in BP from baseline to Week 8 in patients who are adding indapamide prolonged‐release formulation to perindopril (free‐combination therapy) compared with perindopril alone (monotherapy). Perindopril and indapamide were largely studied alone or in combination [7, 8, 12, 13, 14, 15, 16], but no data are available comparing the prolonged‐release formulation of indapamide in combination with perindopril to perindopril alone. A secondary methodological objective was to assess alternative statistical methods and population definitions for identifying the antihypertensive effects of free‐combination therapy versus monotherapy. Specifically, we analyzed BP measures collected in routine practice via EMRs in the UK's Clinical Practice Research Datalink (CPRD Aurum) and implemented several approaches to minimize potential confounding and selection bias when comparing antihypertensive strategies.

## 2 Materials and Methods

### 2.1 Target Trial Specification

To estimate the effect of adding indapamide prolonged‐release formulation to perindopril on BP using observational data, we first specified the target trial (Table 1). Briefly, eligibility criteria for the trial population would have been age ≥ 18 years, diagnosis of primary hypertension at baseline (systolic blood pressure [SBP] ≥ 145 mmHg and < 160 mmHg in the 2 weeks before baseline) despite stable therapy with perindopril 5 mg for ≥ 4 weeks, and no use of any other antihypertensive treatment in the preceding 4 weeks. To exclude transient limited increase of SBP and ensure inclusion of true uncontrolled hypertension, 145 mmHg was selected as the threshold for uncontrolled systolic hypertension (typically defined by SBP ≥ 140 mmHg in clinical practice). Patients with moderate to severe uncontrolled hypertension (SBP ≥ 160 mmHg and diastolic blood pressure [DBP] ≥ 100 mmHg) would have been excluded for ethical reasons, to avoid enrolling patients who could benefit from a more aggressive therapy (particularly if they were randomized to the arm with no addition of indapamide prolonged‐release). Patients would have been randomized either to continue receiving perindopril 5 mg (control arm) or to receive indapamide prolonged‐release 1.5 mg in addition to perindopril 5 mg (free‐combination arm). The primary estimand [18] of interest would have been the difference in mean change from baseline in SBP after 8 weeks of perindopril 5 mg/indapamide prolonged‐release 1.5 mg compared with perindopril 5 mg monotherapy in patients with hypertension not controlled on perindopril 5 mg monotherapy, assuming no change in assigned therapy.

**TABLE 1:** Target trial specification and emulation.

	Target trial	Emulated trial
Aim	*Primary objective:* To show the superiority of indapamide prolonged‐release 1.5 mg in addition to perindopril 5 mg compared with perindopril 5 mg alone in decreasing BP in patients with SBP ≥ 145 mmHg	*Primary objective:* Same
		*Secondary objective:* To assess alternative statistical methods and population definitions for identifying the antihypertensive effects of free‐combination therapy versus monotherapy
Eligibility criteria	Inclusion criteria	Inclusion criteria
	Age ≥ 18 years	Age ≥ 18 years
	Diagnosis of primary hypertension, and the following measurements: SBP ≥ 145 mmHg and < 160 mmHg and DBP < 100 mmHg^a^	Diagnosis of primary hypertension, defined by the presence of a medical code for primary hypertension any time before baseline or by 2 successive records of SBP ≥ 140 mmHg and/or DBP ≥ 90 mmHg at distinct dates within 6 months before baseline Last record within 2 weeks before baseline of SBP ≥ 145 mmHg and < 160 mmHg and DBP < 100 mmHg
	Received stable treatment with perindopril 5 mg for ≥ 4 weeks before baseline	Received a prescription of perindopril 4 or 5 mg daily at a stable dose for ≥ 4 weeks before baseline Prescription of perindopril 4 or 5 mg during the year before baseline Initiated indapamide prolonged‐release 1.5 mg in addition to the ongoing exposure to perindopril or received a renewal prescription of perindipril 4 mg or 5 mg
	Did not receive other antihypertensive treatment during the 4 weeks before baseline	Did not receive other antihypertensive treatment during the 4 weeks before baseline Did not receive indapamide during the 12 months before baseline Had ≥ 12 months of continuous enrollment in the data source before baseline Had Hospital Episode Statistics linkage available
	Exclusion criteria	Exclusion criteria
	Diagnosis of secondary hypertension	Diagnosis of secondary hypertension
	Moderate to severe uncontrolled hypertension with all SBP values ≥ 160 mmHg or all DBP values ≥ 100 mmHg recorded at baseline^a^	Moderate to severe uncontrolled hypertension with SBP ≥ 160 mmHg and DBP ≥ 100 mmHg ≥ 1 SBP value < 145 mmHg (indicative of controlled hypertension) if several values were recorded on the date of baseline BP Prescription at baseline of any antihypertensive treatment other than indapamide prolonged‐release 1.5 mg or perindopril 4 or 5 mg, including other dosages of perindopril, identified with record of at least 1 product code
	Cerebrovascular, liver, or renal comorbidities or history of hypokalemia	Cerebrovascular,^b^ liver,^c^ or renal comorbidities^d^ or history of hypokalemia^e^
Treatment strategies	*Indapamide prolonged‐release + perindopril arm:* Addition of indapamide prolonged‐release 1.5 mg to perindopril 5 mg using a single pill combination	*Free‐combination arm:* Addition of indapamide prolonged‐release 1.5 mg to perindopril 4 or 5 mg (bioequivalent doses) in a 2‐pill free combination
	*Perindopril‐alone arm:* Remain on perindopril 5 mg	*Monotherapy arm:* Remain on perindopril 4 or 5 mg
		We considered treatment to be continuous if there was a gap of less than 30 days between successive prescriptions. Overlap of prescription was shifted forward.
Treatment assignment	Patients are randomly assigned to either arm at baseline. Baseline is defined as the beginning of the randomization period (i.e., the time of treatment assignment).	We classified individuals according to the observed treatment strategy at baseline and emulated randomization by matching individuals in the monotherapy arm to those in the free‐combination arm on a 3:1 basis based on the PS. Baseline covariates that remained imbalanced after matching^f^ were adjusted for.
		Patients who initiated indapamide prolonged‐release during an ongoing episode of perindopril and those who initiated indapamide prolonged‐release at the same time as renewal of a perindopril prescription were included in the free‐combination arm. Baseline in this group was the date of indapamide prolonged‐release prescription. Patients who received a renewal prescription of perindopril 5 or 4 mg without adding any other antihypertensive treatments were assigned to the monotherapy arm. Baseline in this group was the date of perindopril prescription renewal.
		Eligibility criteria were met several times: patients were included in a treatment group (as individuals) each time they were eligible and aligned with study treatment strategies. A single patient could be included and contribute to the analysis as several individuals with different baselines and, potentially, different exposures (i.e., multiple eligibility) [17].
Follow‐up	Patients are followed from treatment assignment to 12 weeks afterward.	Patients were followed from treatment assignment until the end of practice data, transfer out of the practice, any change in assigned treatment (including addition of other antihypertensive treatments or increased dose of indapamide prolonged‐release or perindopril), discontinuation of treatment of interest, death, or 1 year after baseline, whichever occurred first.
	Patients are monitored with 1 visit and BP measurement after 4 and 8 weeks.	
Outcome	Change in SBP (primary outcome) or DBP (secondary outcome) between baseline and Week 8.	Same
		Baseline BP was defined as the measurement taken within 2 weeks before or at baseline. Outcome BP was defined as the measurement taken between Weeks 4 and 24 after baseline that was closest to Week 8 after baseline.
		When multiple BP measurements were recorded on the same day, the mean value was used.
Estimand	The primary estimand of interest is the difference in mean change from baseline in sitting SBP after 8 weeks of indapamide prolonged‐release 1.5 mg in addition to perindopril 5 mg compared with perindopril 5 mg monotherapy in patients with hypertension not controlled (SBP 145–159 mmHg and DBP < 100 mmHg) on perindopril 5 mg monotherapy, assuming no other antihypertensive concomitant treatments were taken and assuming no switch to other therapy following premature treatment discontinuation.	Same
	The ICEs that are considered in the estimand definitions are: premature treatment discontinuation or dose increase or use of any other antihypertensive treatment.	
Statistical analysis	The change in mean BP is estimated via linear regression (ANCOVA).	Same
		Additional adjustment occurred for imbalanced baseline variables.
	Patients deviating from the assigned treatment strategy are censored and assigned a missing outcome. Missing outcomes in case of ICEs are imputed with the baseline BP value assuming no treatment benefit.	Patients deviating from the assigned treatment strategy, including addition of or switch to other antihypertensive treatments, increased dose, or discontinuation of assigned treatment, were censored. Missing outcomes in case of such ICEs were imputed with the baseline BP value assuming no treatment benefit.
	Other missing data (missing outcomes in compliant patients and baseline variables in any patient) are handled via multiple imputation under the assumption that they are MAR.	Missing outcomes for patients who remained treated with the baseline strategy until 24 weeks were handled via multiple imputation, under the assumptions that they are MAR and that these individuals would have had similar effectiveness outcomes as individuals from their treatment group.

Abbreviations: ANCOVA, analysis of covariance; ASD, absolute standardized difference; BMI, body mass index; BP, blood pressure; CPRD, clinical practice research datalink; DBP, diastolic blood pressure; HES, hospital episode statistics; ICD‐10, international classification of diseases, 10th revision; ICE, intercurrent event; IPCW, inverse probability of censoring weighting; MAR, missing at random; PS, propensity score; SBP, systolic blood pressure.

^a^ Individuals with SBP values ≥ 160 mmHg or all DBP values ≥ 100 mmHg would have been excluded in a randomized controlled trial for ethical reasons, to avoid including patients who could benefit from a more aggressive therapy, particularly if they were randomized to the arm with no addition of indapamide prolonged‐release.

^b^ Cerebrovascular comorbidity was defined as history of hospitalization for cerebrovascular disease (ischemic stroke, cerebral hemorrhage, and/or transient ischemic attack) or severe heart disease (shock, including cardiogenic shock; myocardial infarction; unstable angina pectoris; and/or coronary revascularization) that was defined by ≥ 1 primary ICD‐10 diagnosis code for cerebrovascular disease or severe heart disease recorded in HES data in the 12 months before or at baseline.

^c^ Liver comorbidity was defined as history of hospitalization for severe liver disease that was defined by ≥ 1 primary liver disease ICD‐10 diagnosis code recorded in HES data 12 months before or at baseline.

^d^ Renal comorbidity was defined as having ≥ 1 severe or moderate renal failure diagnosis medical code recorded in the 12 months before or at baseline, or with ≥ 1 hospitalization for renal failure defined by ≥ 1 primary ICD‐10 diagnosis code registered in HES data in the12 months before or at baseline or with a glomerular filtration rate < 60 mL/min/1.73 m^2^ according to their most recent glomerular filtration rate recorded in the 12 months before or at baseline.

^e^ History of hypokalemia was defined as having ≥ 1 hypokalemia diagnosis code recorded in CPRD data in the 12 months before or at baseline, or a serum potassium < 3.6 mmol/L based on the most recent record in the 12 months before or at baseline, or a prescription of potassium supplementation identified with ≥ 1 record of product code of potassium supplementation in the 12 months before or at baseline.

^f^ eAppendix B presents the covariates evaluated at baseline and included in the PS model.

### 2.2 Target Trial Emulation

#### 2.2.1 Data Sources

We emulated this target trial via a matched cohort design (Table 1) [10, 11] using the UK's CPRD Aurum database, which collects anonymized EMR data from general practitioners and, as of April 2021, included data for approximately 13 million patients registered at approximately 1400 practices [19]. The study was approved by the CPRD's Independent Scientific Advisory Committee (ISAC) in March 2021 (protocol number 20_000194). eAppendix A describes CPRD Aurum in detail. For assessment of covariates, primary care data from CPRD Aurum were linked to 2 additional data sources: Index of Multiple Deprivation (IMD) for socioeconomic data and the Hospital Episode Statistics Admitted Patient Care (HES APC) for diagnoses recorded in hospitals.

#### 2.2.2 Study Population

Patients were identified between January 2000 and March 2020 and were followed for up to 12 months. To emulate the target trial eligibility criteria, a diagnosis of primary hypertension was defined by the presence of a medical code for primary hypertension any time before baseline or by 2 successive records of elevated BP (SBP ≥ 140 mmHg and/or DBP ≥ 90 mmHg) at distinct dates within 6 months before baseline. Table 1 presents the inclusion and exclusion criteria. Eligible patients either initiated indapamide prolonged‐release 1.5 mg in addition to an ongoing exposure to perindopril arginine 5 mg (or an equivalent dose of perindopril *tert*‐butylamine 4 mg) daily (free‐combination initiation) or received a renewal prescription of perindopril 4 mg or 5 mg daily (monotherapy continuation) the same day or in the 2 weeks after an SBP measurement of ≥ 145 mmHg and < 160 mmHg. Perindopril 5 mg is rarely used in the UK, and perindopril 4 and 5 mg are bioequivalent [20]; thus, these 2 dosages were interchangeably used. Study baseline was the date of the new indapamide prolonged‐release prescription for the free‐combination arm or renewed perindopril prescription for the monotherapy arm. Eligibility was assessed at the study baseline. To properly evaluate baseline variables, patients were required to have ≥ 12 months of continuous enrollment in CPRD Aurum before baseline and linkage with HES APC data.

Patients were followed from treatment assignment until the end of practice data, transfer out of practice, any change from assigned treatment including addition of other antihypertensive treatments, increased dose, or discontinuation, death, or 1 year after baseline, whichever occurred first.

#### 2.2.3 Treatment Groups

**FIGURE 1:**
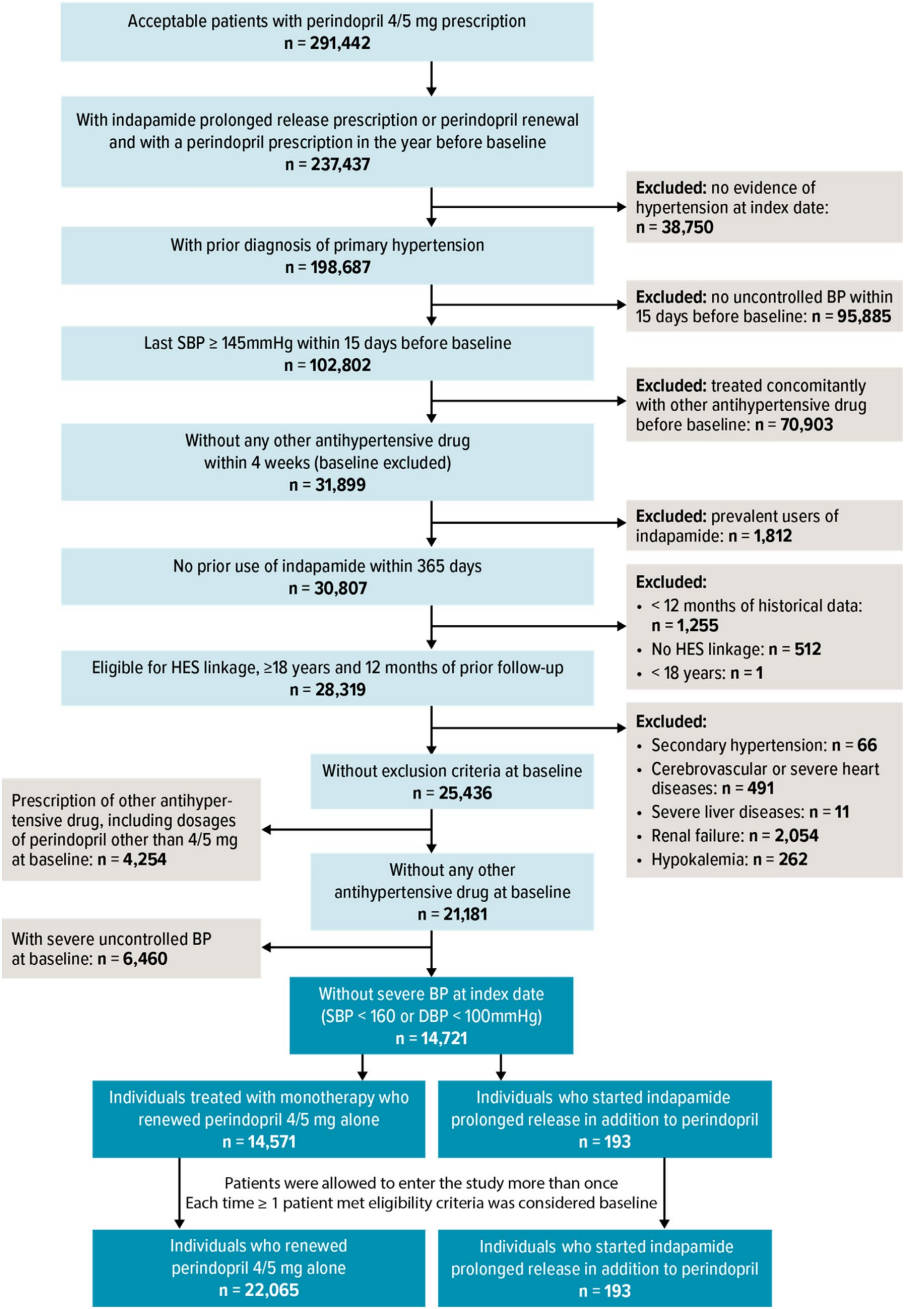
Study population flow chart. BP = blood pressure; DBP = diastolic blood pressure; HES = Hospital Episode Statistics; HT = hypertension; SBP = systolic blood pressure. eTable D1 presents the number of inclusions in the perindopril arm and/or free combination arm in the matched cohort.

Treatment strategies were the same as in the target trial. Eligibility criteria could be met several times (see Figure 1) and patients were included in a treatment group (as individuals) each time they were eligible and aligned with study treatment strategies. Therefore, a single patient could be included in and contribute to the analysis as several individuals with different baselines and, potentially, different exposures (i.e., multiple eligibility) [17]. Patients were matched in a 3:1 ratio (monotherapy: free combination) on a propensity score (PS) that accounted for sociodemographic characteristics, hypertension history, practice‐specific variables, treatment history, cardiovascular history, use of healthcare resources, comorbidities, and concomitant therapies (Table 1). Comorbidities selected to balance the study population were the most medically relevant confounders on SBP measurement. The absolute standardized difference (ASD) was used to assess covariate balance between treatment groups before and after matching. Variables with an ASD ≥ 0.1, indicative of remaining imbalance in the matched population, were adjusted on in the final treatment‐effect estimation model (see eAppendix B).

#### 2.2.4 Estimand

The estimand [18] was the effectiveness estimate for free combination compared with monotherapy in the treated population (i.e., average treatment effect in the treated population [ATT]) with SBP ≥ 145 mmHg, during ongoing exposure to the assigned therapy. The primary outcome of the emulated trial was change in SBP between baseline and Week 8. To account for actual frequency of BP reports in EMRs, the BP measurement closest to Week 8 in the interval of Weeks 4–24 after baseline was used to maximize the number of patients with an available BP outcome. Intercurrent events (ICEs) considered for this estimand were treatment changes including prescription of other antihypertensive treatments, increased dose, and discontinuation of assigned treatment.

### 2.3 Primary Analysis

The treatment effect—the mean difference in SBP (primary outcome) between baseline and Week 8 for matched treatment groups—was estimated by a linear regression (analysis of covariance). ICEs were handled using a hypothetical strategy of treatment failure, and outcomes of patients with ICEs (and no BP record before ICE occurrence) were imputed using the baseline BP value and assuming no treatment benefit or worsening (missing not at random assumption) [21]. Thus, the treatment effect reflected a lack of benefit for the patient without considering the effect of other therapies following premature treatment change.

Missing outcomes for patients treated with the baseline strategy until Week 24 and without ICEs were handled via multiple imputation, assuming that these patients would have had similar effectiveness outcomes as patients from their treatment group (missing at random [MAR] assumption) [21].

To account for the effect of multiple eligibility and matching on variance estimation, 1000 bootstrapped samples were generated to estimate the 95% confidence intervals (CIs). Estimates and variance estimates were combined using Rubin rules [22].

### 2.4 Secondary and Sensitivity Analyses

As a secondary outcome, the mean difference in DBP from baseline to Week 8 between matched treatment groups was estimated with a similar approach as in the primary analysis. A secondary methodological objective of the study was to assess several definitions of the study population and different analytical approaches to the study of BP in CPRD Aurum. Specifically, a complete‐case analysis was conducted to assess the impact of missing data, and standardized mortality ratio (SMR) weighting [23] was used instead of PS matching. Additional study populations, including a “broader population” of patients with more severe hypertension and a “restricted uncontrolled population” of patients with 2 successive measurements of SBP ≥ 145 mmHg, were explored. Multiple sensitivity analyses were conducted evaluating different definitions of continuous treatment, the impact of restricting the free‐combination arm to patients initiating indapamide on the same date their perindopril prescription was renewed, the potential for bias in the comparison of new users of free‐combination therapy with prevalent users of monotherapy due to differences in disease course between treatment arms using a time‐conditional PS approach, and the impact of extending the window for SBP outcome assessment to 52 weeks after baseline. eAppendix C describes these additional analyses in detail.

## 3 Results

### 3.1 Study Population

Applying the inclusion and exclusion criteria to the 291 442 users of perindopril 4/5 mg identified between January 2000 and March 2020 resulted in 22 065 individuals (corresponding to 14 571 unique patients) assigned to the monotherapy arm and 193 individuals (all unique) assigned to the free‐combination arm (Figure 1).

Before PS matching, the overall included population was 54.3% male (monotherapy: 54.3%; free combination: 53.4%); mean (± standard deviation [SD]) age was 64.5 years (±12.4), and individuals receiving free combination were younger than those receiving monotherapy (61.5 vs. 64.6 years, respectively). Most individuals were aged < 75 years (monotherapy: 77.3%; free combination: 84.3%); most were White (monotherapy: 92.1%; free combination: 92.2%), followed by Asian (3.1% and 3.1%, respectively). Additional demographics, clinical characteristics, and details of medical and treatment history, before and after PS matching, are summarized in eAppendix D (eTables D2–D8).

All 193 eligible individuals in the free‐combination arm were successfully matched to 579 individuals in the monotherapy arm. In this matched cohort, 1 patient contributed as an eligible individual to both the free‐combination arm and the monotherapy arm, 192 patients contributed uniquely and only once as individuals to the free‐combination arm, and 548 patients contributed as 578 individuals (15 patients contributed twice) only to the monotherapy arm (eTable D1). Covariates at baseline were generally well balanced after matching (Table 2 and eTables D2–D8, eAppendix D). Some covariates that remained imbalanced after matching (see eFigure D1, eAppendix D) had an ASD that exceeded 0.1, but all were close to 0.1. These covariates were added in the final treatment‐effect estimation model to account for the remaining confounding bias. Mean (±SD) duration of follow‐up was 7.6 months (±4.6) for the overall matched cohort, 6.2 months (±4.7) for the free‐combination arm, and 8.0 months (±4.5) for the monotherapy arm.

**TABLE 2:** Baseline characteristics of study population after propensity score matching.

	Monotherapy (*N* = 579)	Free combination (*N* = 193)	ASD
Gender, *n* (%)			
Male	294 (50.8)	103 (53.4)	
Female	285 (49.2)	90 (46.6)	0.052
Age (years), mean ± SD	61.2 ± 12.1	61.5 ± 12.8	0.025
Ethnicity, *n* (%)			
White	532 (91.9)	178 (92.2)	0.013
Mixed/multiple ethnic groups	< 5^a^	—	0.083
Asian or Asian British	10 (1.7)	6 (3.1)	0.090
Black/African/Caribbean/Black British	9 (1.6)	< 5^a^	**0.102**
Other	< 5^a^	< 5^a^	0.059
Missing	25 (4.3)	7 (3.6)	0.035
Body mass index, class, *n* (%)			
Underweight	—	< 5^a^	**0.102**
Normal	119 (20.6)	39 (20.2)	0.009
Overweight	210 (36.3)	72 (37.3)	0.021
Obesity	74 (12.8)	25 (13.0)	0.005
Severely obese	69 (11.9)	21 (10.9)	0.033
Missing	107 (18.5)	35 (18.1)	0.009
Body mass index (kg/m^2^), mean	29.1 ± 5.6	28.9 ± 5.2	0.034
SBP at baseline (mmHg)			
Mean ± SD	152.5 ± 5.3	152.8 ± 4.7	0.053
Median	152.0	152.0	
Q1; Q3	149.0; 156.0	150.0; 156.0	
DBP at baseline (mmHg)			0.010
Mean ± SD	86.2 ± 8.7	86.1 ± 9.2	
Median	88.0	88.0	
Q1; Q3	80.0; 92.0	80.0; 92.0	
Severity of hypertension, *n* (%)			
SBP 145–159 mmHg and/or DBP 90–99 mmHg	558 (96.4)	186 (96.4)	0.000
SBP 160–179 mmHg and/or DBP 100–119 mmHg^b^	18 (3.1)	7 (3.6)	0.029
SBP ≥ 180 mmHg and/or DBP ≥ 120 mmHg^b^	< 5^a^	—	**0.102**
Number of previous antihypertensive treatments in the year before baseline (perindopril excluded), mean ± SD	0.3 ± 0.6	0.3 ± 0.6	0.014
0	442 (76.3)	143 (74.1)	0.052
1	99 (17.1)	39 (20.2)	0.080
2	33 (5.7)	10 (5.2)	0.023
3	< 5^a^	< 5^a^	0.022
≥ 4	< 5^a^	—	0.059
Cardiovascular comorbidities, *n* (%)			
Stroke	82 (14.2)	26 (13.5)	0.020
Myocardial infarction	< 5^a^	< 5^a^	**0.112**
Heart failure	< 5^a^	< 5^a^	0.000
Periphery artery disease	22 (3.8)	5 (2.6)	0.069
Coronary artery disease	29 (5.0)	8 (4.1)	0.041
Cardiac revascularization	< 5^a^	< 5^a^	0.026
Left ventricular hypertrophy	8 (1.4)	< 5^a^	0.032

*Note:* ASDs considered as imbalanced (i.e., ≥ 0.1 [24]) are shown in bold. Any covariates that remained imbalanced (ASD ≥ 0.1) after PS matching were included in the final treatment‐effect model to consider residual confounding. eAppendix D summarizes in detail the population characteristics before and after PS matching.

Abbreviations: ASD, absolute standardized difference; CPRD, clinical practice research datalink; DBP, diastolic blood pressure; PS, propensity score; SBP, systolic blood pressure; SD, standard deviation.

^a^ Exact data not provided, as the minimum cell count must be 5 in accordance with CPRD data governance.

^b^ Patients were excluded if all their SBP measurements reported at baseline were ≥ 160 mmHg or all DBP ≥ 100 mmHg. If there was at least 1 SBP measurement < 160 mmHg or < 100 mmHg, the patient was included. As a result, some patients had their mean SBP value ≥ 160 mmHg and/or mean DBP ≥ 100 mmHg despite the eligibility criteria.

### 3.2 Primary Analysis

**FIGURE 2:**
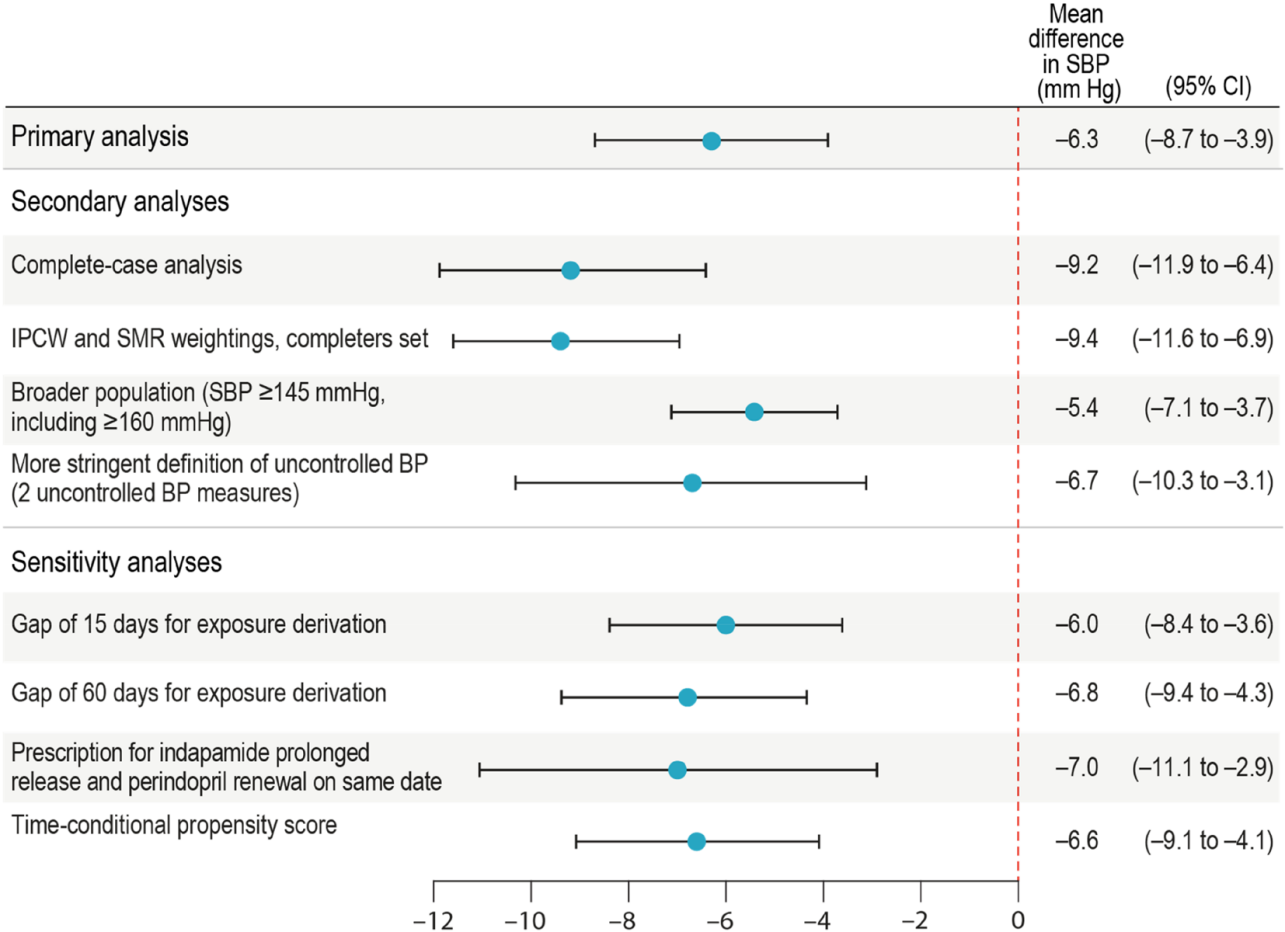
Forest plot of the primary outcome: Primary, Secondary, and Sensitivity Analyses. BP = blood pressure; CI = confidence interval; IPCW = inverse probability censoring weighting; SBP = systolic blood pressure; SMR = standardized mortality ratio.

In the primary analysis, time to the SBP measurement used as an outcome is described in eTable D9. After PS matching, 58 individuals (30.1%) in the free‐combination arm and 89 (15.4%) in the monotherapy arm changed or stopped their original treatment (before having BP recorded between 4 and 24 weeks); outcome BP was imputed by baseline value for these individuals. Nineteen individuals (9.8%) in the free‐combination arm and 151 individuals (26.1%) in the monotherapy arm without outcome or ICEs between Weeks 4–24 had their outcome imputed using multiple imputation. At Week 8, mean (±SD) reduction in SBP values was −3.3 mmHg (±12.8) for individuals receiving monotherapy and −9.9 mmHg (±12.2) for individuals receiving free combination; mean (±SD) reductions in DBP values were −1.6 mmHg (±8.4) and −3.8 mmHg (±8.0), respectively (Table 3). Once adjusted for final imbalanced covariates, the addition of indapamide prolonged‐release to perindopril led to an additional reduction of −6.3 mmHg (95% CI, −8.7 to −3.9) in SBP (Figure 2) and −2.3 mmHg (95% CI, −3.7 to −0.8) in DBP compared with the continuation on perindopril monotherapy (Table 3).

**TABLE 3:** SBP and DBP changes between baseline and Week 8: Primary analysis population.

	Monotherapy (*N* = 579)	Free combination (*N* = 193)
*SBP*		
SBP at baseline (mmHg)		
Mean ± SD	152.5 ± 5.3	152.8 ± 4.7
Median	152.0	152.0
Q1; Q3	149.0; 156.0	150.0; 156.0
Min; Max	145; 188	145; 170
SBP at Week 8 (mmHg)		
Mean ± SD	149.2 ± 12.4	142.8 ± 12.0
Median	149.0	145.0
Q1; Q3	142.0; 155.0	135.0; 152.0
Min; Max	106; 218	105; 172
SBP change between baseline and Week 8 (mmHg)		
Mean ± SD	−3.3 ± 12.8	−9.9 ± 12.2
Median	−2.0	−8.0
Q1; Q3	−10.0; 0.8	−18.0; 0.0
Min; Max	−48; 68	−50; 17
Comparison between treatment groups of SBP change (mmHg), estimate (95% CI)^a^		−6.3 (−8.7 to −3.9)
*DBP*		
DBP at baseline (mmHg)		
Mean ± SD	86.2 ± 8.7	86.1 ± 9.2
Median	88.0	88.0
Q1; Q3	80.0; 92.0	80.0; 92.0
Min; Max	51; 110	60; 102
DBP at Week 8 (mmHg)		
Mean ± SD	84.6 ± 8.9	82.2 ± 9.0
Median	85.0	82.0
Q1; Q3	80.0; 90.0	78.0; 88.0
Min; Max	51; 120	58; 104
DBP change between baseline and Week 8 (mmHg)		
Mean ± SD	−1.6 ± 8.4	−3.8 ± 8.0
Median	0.0	−1.6
Q1; Q3	−5.9; 2.0	−8.0; 0.0
Min; Max	−37; 48	−30; 36
Comparison between treatment groups of DBP change (mmHg), estimate (95% CI)^a^		−2.3 (−3.7; −0.8)

Abbreviations: CI, confidence interval; DBP, diastolic blood pressure; SBP, systolic blood pressure; SD, standard deviation.

^a^ Bootstrapped 2‐sided 95% CI of the estimate, obtained combining estimates and standard errors using Rubin's rule.

### 3.3 Secondary Analyses

When considering the subpopulation of matched completers (patients with an outcome recorded while treated with baseline strategy; *N* = 460), the additional SBP reduction was −9.2 mmHg (95% CI, −11.9 to −6.4) for free‐combination compared with monotherapy (Figure 2; eTable E1, eAppendix E). When the alternative SMR–ICPW method was applied to completers, the additional SBP reduction was −9.4 mmHg (95% CI, −11.6 to −6.9) (Figure 2; eTable E2, eAppendix E).

Results for the alternative populations were similar to those for the main population (Figure 2; eTable E1, eAppendix E). The analysis of the broader matched population (including more severe patients, with no restriction for those who had all their SBP values ≥ 160 mmHg or DBP ≥ 100 at baseline [*N* = 2332]) yielded an additional SBP reduction of −5.4 mmHg (95% CI, −7.1 to −3.7) with free combination compared with monotherapy. The analysis of the matched population that applied a stringent definition of uncontrolled BP (i.e., the restricted uncontrolled population; see eAppendix C) (*N* = 376) resulted in an additional SBP reduction of −6.7 mmHg (95% CI, −10.3 to −3.1).

### 3.4 Sensitivity Analyses

The sensitivity analyses produced results similar to those from the main analysis (Figure 2). Mean differences in SBP for the comparison of free combination versus monotherapy were in line with results from the main analysis when gaps of 15 days (−6.0 mmHg; 95% CI, −8.4 to −3.6) or 60 days (−6.8 mmHg; 95% CI, −9.4 to −4.3) were used to derive treatment‐exposure duration. When restricting the free‐combination arm to patients with prescriptions for both indapamide prolonged‐release and perindopril renewal on the same date, the difference in SBP between study arms was −7.0 mmHg (95% CI, −11.1 to −2.9). Results from the analysis using time‐conditional PS were similar to those from the main analysis: an additional SBP reduction of −6.6 mmHg (95% CI, −9.1 to −4.1) was observed for free combination compared with monotherapy. Finally, applying a 52‐week window for evaluating the SBP outcome closest to Week 8 resulted in a similar estimate for SBP reduction as in the main analysis (−6.5 mmHg; 95% CI, −8.8 to −4.1). In this analysis, the number of patients with a BP outcome available while on their original treatment increased from 116 to 126 for the free‐combination arm and from 339 to 465 for the monotherapy arm.

## 4 Discussion

Results of this study reveal that beginning the addition of indapamide prolonged‐release 1.5 mg to perindopril 4/5 mg for patients with an SPB ≥ 145 mmHg was associated with a clinically relevant decrease in SBP. In the primary analysis, the addition of indapamide prolonged‐release to perindopril led to an additional SBP reduction of −6.3 mmHg (95% CI, −8.7 to −3.9) and an additional reduction of −2.3 mmHg (95% CI, −3.7 to −0.8) in DBP at Week 8 compared with perindopril monotherapy. Results are consistent with previous studies comparing the antihypertensive effects of combination regimens to monotherapy and with current guidelines on the management of hypertension [9, 25, 26, 27, 28]. Results are also within the range of results from placebo‐controlled clinical trials evaluating the antihypertensive effects of indapamide or indapamide plus perindopril, which have reported decreases in SBP between 5.6 and 15 mmHg [29].

In the analysis of matched completers, the additional reduction in SBP achieved with free combination was greater (−9.2 mmHg [95% CI, −11.9 to −6.4]) than in the primary analysis. The results of the complete‐case analysis can only be applied to the entire study population under the assumption that data are missing completely at random. To relax such strong assumptions, we imputed baseline values to the outcomes of patients who changed their assigned treatment (30.1% in the free‐combination arm and 15.4% in the monotherapy arm). Such single imputation assumes that the reason for changing treatment was no improvement in SBP and provides a conservative effect estimate. For patients with missing outcomes and no treatment change, these were imputed via multiple imputation, which assumes that the outcome is MAR. A third approach to deal with missing outcomes comprised the use of inverse probability weighting, which weights completers to account for similar individuals without outcome records or with ICEs who are excluded from the analysis, assuming that missingness is at random conditional on the covariates used to estimate the weights (in our case, the same used for the PS model). Additionally, a wider time‐window for SBP outcome assessment, using the closest SBP value up to 8 weeks over a period of 4–52 weeks after baseline was used to decrease the number of missing outcomes. This wider period to ascertain the outcome assumes that it does not introduce measurement error in the outcome. All assumptions to deal with missing outcomes are unverifiable, but the consistency of the results under all different approaches (and assumptions) is reassuring for a true effect of the free‐combination on SBP compared with monotherapy. The potential for regression to the mean was accounted for by including baseline SBP in the final model.

Results of the primary analysis were consistent with results from samples defined using different eligibility criteria, including those defined by moderate to severe uncontrolled hypertension. Results stratified by calendar year and by age were consistent with the main analysis (eTable E3). In the target trial, these individuals would have been excluded to avoid enrolling patients who could potentially benefit from more aggressive therapy. In this observational study, BP outcomes for individuals with more severe uncontrolled hypertension were able to be evaluated with no additional harm. Results of the primary analysis were also consistent with those from sensitivity analyses, including use of time‐conditional PS matching (−6.6 mmHg [95% CI, −9.1 to −4.1]). Alternative methods to adjust for selection bias due to censoring and confounding provided a different estimate of treatment effect (−9.4 mmHg [95% CI, −11.6 to −6.9]) closer to the complete‐case result. Overall, study findings were consistent across alternative methods accounting for the real‐world nature of the data.

As RWD become increasingly relevant in premarketing regulatory decisions, comparative‐effectiveness evidence is needed. Nonetheless, limitations related to the use of an automated healthcare database as a data source and to inconsistent BP recordings in EMRs must be noted. Assessment of SBP at Week 8 as a main outcome in CPRD Aurum was associated with missing data, and some patients discontinued or changed treatment during the outcome assessment time window without necessarily having a BP measurement taken beforehand. To avoid related selection bias, all patients in the matched cohort were analyzed, and imputation strategies accounted for ICEs and missing outcomes, as described above. Furthermore, BP readings were collected from EMRs and were not validated. Therefore, the potential for measurement bias due to measurement error (likely non‐differential) needs to be considered.

Results can be subject to confounding bias and, in particular, to channeling bias, because patients starting combination therapy may be more severe patients than patients remaining treated with perindopril alone. Measured confounders were adjusted for via PS matching. To further control for confounding related to severity, a time‐conditional PS based on duration of perindopril exposure was conducted [30]. Nonetheless, residual confounding due to unmeasured factors like diet or lifestyle may still be present, although we did adjust for body mass index, smoking, and alcohol consumption, which are likely good surrogates of those unmeasured factors. Finally, although the analyses did not account for medication adherence during follow‐up, adherence to perindopril in the year before baseline was taken into account. The occurrence of adverse events was not evaluated because they were out of the scope of this study.

## 5 Conclusions

This comparative‐effectiveness study used RWD from CPRD Aurum to emulate a hypothetical target trial evaluating the effectiveness of adding indapamide prolonged‐release to perindopril for BP reduction in patients with SBP ≥ 145 mmHg. Our data are compatible with a clinically meaningful BP reduction at 8 weeks of therapy due to the addition of indapamide prolonged‐release to perindopril compared with perindopril monotherapy in patients with an SBP ≥ 145 mmHg previously taking perindopril monotherapy. Results were consistent in secondary and sensitivity analyses, showcasing some epidemiological methods for analyzing BP outcomes in CPRD Aurum that may be informative for future research using this data source.

## Funding

This work was supported by Servier.

## Conflicts of Interest

This research and development of this publication were funded by Servier. Céline Darricarrere, Virginie Simon, Emmanuelle Jacquot, and Dominique Procureur were employees of Servier when this research was conducted. Morgane Ballon and Marie Mangin were full‐time consultants for Servier when this research was conducted. Jaume Aguado, Manel Pladevall‐Vila, and Xabier Garcia de Albeniz Martinez are employees of RTI Health Solutions, which received research funding to collaborate on this study.

## Supporting information


**Data S1:** Supplementary appendix.

## Data Availability

The study data are not available for replication to protect patient confidentiality.
