# Supplementary material for: Effectiveness of Indapamide Prolonged‐Release and Perindopril Versus Perindopril Monotherapy for Treated Uncontrolled Hypertension: A Target Trial Emulation
Source: Pharmacoepidemiol Drug Saf. 2026 Feb 8;35(2):e70295. doi: 10.1002/pds.70295 (PMC12884010; doi:10.1002/pds.70295)
Supplement: Supplementary file 1 — Data S1: Supplementary appendix. [file PDS-35-e70295-s001.docx]

Effectiveness of Indapamide Prolonged-Release and Perindopril Versus Perindopril Monotherapy for Treated Uncontrolled-Hypertension: A Target Trial Emulation

**Authors:**

Céline DARRICARRERE^a^ *, [0000-0002-5306-6906](https://orcid.org/0000-0002-5306-6906)

Virginie SIMON^a^ *^,^**, [0009-0003-2757-8697](https://orcid.org/0009-0003-2757-8697)

Manel PLADEVALL-VILA^b,c^, [0000-0002-9359-6055](https://orcid.org/0000-0002-9359-6055)

Emmanuelle JACQUOT ^a^ **

Morgane BALLON^d^, [0000-0003-0584-2795](https://orcid.org/0000-0003-0584-2795)

Marie MANGIN^d^

Dominique PROCUREUR^e^

Jaume AGUADO^b^, [0000-0002-9575-0212](https://orcid.org/0000-0002-9575-0212)

Xabier GARCIA DE ALBENIZ MARTINEZ^b^, [0000-0002-9814-2343](https://orcid.org/0000-0002-9814-2343)

* Co-primary authors.

** Affiliation at the time this research was conducted.

^a^ Servier, Suresnes, France

^b^ RTI Health Solutions, Barcelona, Spain

^c^ The Center for Health Policy and Health Services Research, Henry Ford Health System, Detroit, Michigan, USA

^d^ IT&M Stats, Neuilly-sur-Seine, France

^e^ R&D Life Cycle Management, Servier, Suresnes, France

**Corresponding author:** Céline Darricarrere, Servier, 50 Rue Carnot, Suresnes, France 92284; celine.darricarrere@gmail.com; +01.55.72.60.00

Contents

[eAppendix A: Data Source 5](#_Toc212458769)

[CPRD Aurum 5](#_Toc212458770)

[Data Linkage 5](#_Toc212458771)

[References 7](#_Toc212458772)

[eAppendix B: Propensity Score Modelling And Matching 8](#_Toc212458773)

[eTable B1. Propensity Score Model 10](#_Toc212458774)

[Definition of Logit-PS–Matched Cohorts 14](#_Toc212458775)

[Evaluation of Matching Quality 14](#_Toc212458776)

[eFigure B1. Logit-PS Density Before and After Matching 15](#_Toc212458777)

[References 16](#_Toc212458778)

[eAppendix C: Secondary and Sensitivity Analyses 17](#_Toc212458779)

[Secondary Analyses 17](#_Toc212458780)

[Sensitivity Analyses 19](#_Toc212458781)

[References 20](#_Toc212458782)

[eAppendix D: Population Characteristics 21](#_Toc212458783)

[eTable D1. Correspondence Between Patients and Individuals](#_Toc212458784)^[a](#_Toc212458784)^ [in the Matched Cohort, by Treatment Group (Monotherapy Arm and Free-Combination Arm) 21](#_Toc212458784)

[eTable D2. Baseline Characteristics, Main Study Population, Before and After Matching 22](#_Toc212458785)

[eTable D3. History of Hypertension, Main Study Population, Before and After Matching 24](#_Toc212458786)

[eTable D4. Blood Pressure Records, Practice-Related Variables, Main Study Population, Before and After Matching 27](#_Toc212458787)

[eTable D5. Antihypertensive Treatment Patterns, Main Study Population, Before and After Matching 28](#_Toc212458788)

[eTable D6. Comorbidities, Main Study Population, Before and After Matching 30](#_Toc212458789)

[eTable D7. Concomitant Treatment, Main Study Population, Before and After Matching 31](#_Toc212458790)

[eTable D8. Use of Healthcare Resources, Main Study Population, Before and After Matching 32](#_Toc212458791)

[eTable D9. Timing of SBP Measurement Among Patients With an SBP Measurement While Treated With Baseline Strategy (i.e., “Completers”) 33](#_Toc212458792)

[eFigure D1. Absolute Standardized Differences Before and After Matching, Main Study Population 34](#_Toc212458793)

[eAppendix E: Additional Outcomes in Alternative Populations and With Alternative Analyses 36](#_Toc212458794)

[eTable E1. Systolic Blood Pressure Change Between Baseline and Week 8: Alternative Populations 36](#_Toc212458795)

[eTable E2. Systolic Blood Pressure Change Between Baseline and Week 8: IPCW and SMR Weightings, Completers (N = 11,427) 39](#_Toc212458796)

[eTable E3. Systolic Blood Pressure Change Between Baseline and Week 8: IPCW and SMR Weightings, Completers (N = 11,427) 40](#_Toc212458797)

# eAppendix A: Data Source

## CPRD Aurum

The data source of this study was the Clinical Practice Research Datalink (CPRD Aurum) primary care database. In April 2021, approximately 13 million patients were registered at about 1,400 currently contributing practices (excluding deceased patients and those who transferred out) (CPRD Aurum, 2021). Contributing practices were mostly based in England (99%), but some of them are also based in Northern Ireland (1%) (CPRD Aurum, 2021).

CPRD Aurum routinely collects anonymized electronic health record data from general practitioners (GP) who have agreed at a practice level to provide data on a monthly basis (Wolf et al., 2019). In the United Kingdom (UK), GPs are the primary point of contact for nonemergency healthcare and for referrals of their patients to specialists or to hospitals. They record patient demographics, diagnoses, symptoms, tests, medical history, and drug prescriptions—as well as some lifestyle information and behavioral factors—using Emis^®^ GP software (Wolf et al., 2019), from which CPRD retrieves data. All patients registered within a practice are included in the dataset unless requested otherwise. However, it is impossible to identify patients who move to another practice.

## Data Linkage

For this study, the CPRD Aurum primary care data were linked to the Hospital Episode Statistics (HES) for a subset of patients in England. The HES contain National Health Service (NHS) data related to activities of UK healthcare providers across 5 main patient groups; Admitted Patient Care (APC) data reflect inpatient and day case admissions to hospital.

HES APC contains details of all admissions to or attendances at English NHS healthcare providers. It includes private patients treated in NHS hospitals, patients residing outside England, and care delivered by treatment centers (including those in the independent sector) funded by the NHS. All NHS healthcare providers in England—including acute hospital trusts, primary care trusts, and mental health trusts—submit data (CPRD Aurum, 2022). HES APC data include the complete set of hospital episode information (admission and discharge dates, diagnoses [identifying primary diagnosis], specialists seen, and procedures undertaken) for each linked patient with a hospitalization record. In addition, augmented care data (intensive and/or high dependency levels of care) and maternity data are available (CPRD Aurum, 2022). The latest release of HES APC data (set 21) covered the period from April 1997 to October 2020 and was used in this study. More than 90% of patients from CPRD Aurum could be linked to an NHS identifier in the HES APC data (CPRD Aurum, 2022).

The CPRD Aurum primary care data were also linked to the Index of Multiple Deprivation (IMD). The IMD is a measure of relative deprivation based on the patient postcode. This measure can be used as a proxy for sociodemographic and socioeconomic data, which are generally poorly recorded in the primary care data, as they do not directly relate to a patient's care. Because the IMD is reliable over time, the most recent version, which is the 2015 English IMD, was used. This index is based on a combination of 7 distinct domains of deprivation:

- Income deprivation
- Employment deprivation
- Education, skills, and training deprivation
- Health deprivation and disability
- Crime
- Barriers to housing and services
- Living environment deprivation

Each domain index can itself be a composite score derived from 2 or more subdomain indicators. The IMD is a composite calculated as the weighted sum of the domain indices. Data are provided as quintiles or deciles of the deprivation score or rank to prevent disclosure of patient or practice area; in this study, quintiles were used.

## References

CPRD Aurum. CPRD Aurum April 2021 data set, Version 2021.04.001. <https://doi.org/10.48329/8pm6-4q84>. Accessed 29 November 2022.

Wolf A, Dedman D, Campbell J, Booth H, Lunn D, Chapman J, Myles P. Data resource profile: Clinical Practice Research Datalink (CPRD) Aurum. Int J Epidemiol. 2019 Dec 1;48(6):1740-1740g. doi: 10.1093/ije/dyz034.

CPRD Aurum. CPRD linked data. 2022. <https://cprd.com/cprd-linked-data>. Accessed 29 November 2022.

# eAppendix B: Propensity Score Modelling And Matching

Propensity score (PS) matching was used to adjust confounding factors in order to emulate the random assignment of strategies at baseline and to ensure comparability of the treatment groups. The PS of starting indapamide prolonged-release 1.5 mg on top of perindopril 4 mg or 5 mg (free combination) versus perindopril 4 mg or 5 mg alone (monotherapy) was estimated with a logistic regression on the included population and on each additional population. The estimated PS is the predicted probability of treatment derived from the fitted regression model. The baseline covariates (including systolic and diastolic blood pressure at baseline) that were used in the PS model were selected for their expected confounding effect on the basis of clinical knowledge. For variables that could be expressed both quantitatively and qualitatively, the quantitative form was prioritized (except for body mass index that was categorized); however, in case of convergence or fitting issues, the qualitative form was used. The overlap of PS distributions was assessed visually.

The covariates evaluated at baseline and included in the PS model were as follows (eTable B1 presents the covariates included in the final model and their coefficients):

- Sociodemographic characteristics: Age, sex, ethnicity, socioeconomic status, calendar year at baseline, body mass index, smoking status, alcohol consumption.
- Hypertension history: SBP and DBP at baseline, severity of hypertension at baseline, isolated systolic hypertension at baseline (defined as SBP ≥ 145 mmHg and DBP < 90 mmHg), number of BP records in the year before baseline, proportion of uncontrolled SBP and DBP measures in the year before baseline, duration in months between BP measures, and time since hypertension diagnosis.
- Practice variable: Mean number of BP records per year per patient for the practice.
- Treatment history: Number of antihypertensive treatments and treatment classes in the year before baseline, history of ≥1 thiazide diuretic or angiotensin-converting enzyme inhibitor (perindopril excluded) or angiotensin II receptor blocker prescribed in year before baseline or at baseline, time since first antihypertensive prescription, time since perindopril initiation, adherence to perindopril.
- Cardiovascular history: History of stroke, myocardial infarction, heart failure, peripheral artery disease, coronary artery diseases, cardiac revascularization, and left ventricular hypertrophy.
- Use of healthcare resources: Use of preventive services and number of hospital stays. Comorbidities: Diabetes, dyslipidemia, chronic kidney disease, obstructive bronchopulmonary disorders, metabolic syndrome, atrial fibrillation, valvular heart disease, gout, obstructive sleep apnea, diagnosis from ICD-10 chapter during the year before baseline.
- Concomitant therapies: Lipid lowering therapies, glucose-lowering therapies, antiplatelet therapies, anti-angina therapies, nonsteroidal anti-inflammatory drugs, number of drugs prescribed during the 3 months before baseline.

eTable B1. Propensity Score Model

| **Variable** | **Estimate** | **Standard Error** |
| --- | --- | --- |
| Intercept: TRT=Free combination arm | -23.2110 | 3.0053 |
| YEAR_INCLUSION_CL 1 | 0.1778 | 0.1950 |
| YEAR_INCLUSION_CL 3 | 0.0601 | 0.2150 |
| YEAR_INCLUSION_CL 4 | -0.0423 | 0.2941 |
| GENDER F | 0.0694 | 0.1619 |
| AGE | -0.0211 | 0.00830 |
| ETHNICITY_CL_RECAT Missing | -0.0553 | 0.4020 |
| ETHNICITY_CL_RECAT Non White | -0.1752 | 0.3837 |
| SOCIOECONOMIC STATUS 2nd quintile | 0.1631 | 0.1971 |
| SOCIOECONOMIC STATUS 3rd quintile | -0.4277 | 0.2319 |
| SOCIOECONOMIC STATUS 4th quintile | 0.0107 | 0.2212 |
| SOCIOECONOMIC STATUS 5th quintile (most deprived) | -1.0384 | 0.3307 |
| SOCIOECONOMIC STATUS Missing | 1.4262 | 1.1258 |
| IMPUTED BMI Missing | -0.1918 | 0.2281 |
| IMPUTED BMI Normal | -0.3042 | 0.2055 |
| IMPUTED BMI Obesity | -0.6900 | 0.2387 |
| IMPUTED BMI Severely obese | -0.2515 | 0.2683 |
| IMPUTED BMI Thin | -0.2936 | 1.0660 |
| SBP AT BASELINE | 0.1218 | 0.0178 |
| DBP AT BASELINE | -0.0155 | 0.0140 |
| SMOKER_STATUS_3CL_LAST_IMPUT Current smoker | -0.0941 | 0.2136 |
| SMOKER_STATUS_3CL_LAST_IMPUT Missing | -0.2183 | 0.5654 |
| SMOKER_STATUS_3CL_LAST_IMPUT Never smoker | -0.1334 | 0.1852 |
| DRINK_QUANTI_4CL_LAST_IMPUT Current heavy drinker | 0.0901 | 0.2415 |
| DRINK_QUANTI_4CL_LAST_IMPUT Former drinker | 0.1911 | 0.2563 |
| DRINK_QUANTI_4CL_LAST_IMPUT Missing | 0.0956 | 0.2723 |
| DRINK_QUANTI_4CL_LAST_IMPUT Never drinker | 0.2745 | 0.2293 |
| SEVERITY OF HYPERTENSION Grade 2 or 3 | -0.1847 | 0.4879 |
| ISOLATED SYSTOLIC HYPERTENSION No | 0.7253 | 0.2521 |
| NUMBER OF BP PER PATIENT | 0.0452 | 0.0317 |
| PROPORTION OF UNCONTROLLED SBP | 0.00778 | 0.00444 |
| PROPORTION OF UNCONTROLLED DBP | 0.00246 | 0.00340 |
| IMPUTED DURATION BETWEEN 2BP | -0.1045 | 0.0458 |
| TYPE OF FIRST DIAGNOSTIC OF HYPERTENSION UBP | -0.1067 | 0.2745 |
| TIME SINCE FIRST DIAGNOSTIC OF HYPERTENSION | 0.0187 | 0.0211 |
| HISTORY OF STROKE Yes | 0.6702 | 0.2570 |
| HISTORY OF MYOCARDIAL INFARCTION Yes | -1.0576 | 0.7633 |
| HISTORY OF HEART FAILURE Yes | -1.0461 | 1.0222 |
| HISTORY OF PERIPHERY ARTERY DISEASE Yes | 0.0819 | 0.4774 |
| HISTORY OF CORONARY ARTERY DISEASE Yes | -0.2483 | 0.4379 |
| HISTORY OF CARDIAC REVASCULARIZATION Yes | -0.5166 | 1.0811 |
| HISTORY OF LEFT VENTRICULAR HYPERTROPHY Yes | 0.7117 | 0.7625 |
| DIABETES STATUS Diabetes with HbA1c < 6.5% | 0.4220 | 0.3512 |
| DIABETES STATUS Diabetes with HbA1c >= 6.5% | 0.0199 | 0.3498 |
| DIABETES STATUS Diabetes with unknown HbA1c | 0.3088 | 0.4426 |
| DYSLIPIDEMIA STATUS Yes | 0.2824 | 0.1530 |
| CHRONIC KIDNEY DISEASE Yes | -0.0209 | 0.1826 |
| HISTORY OF OBSTRUCTIVE BRONCHOPULMONARY DISORDERS Yes | -0.3405 | 0.4770 |
| HISTORY OF ATRIAL FIBRILLATION Yes | 0.1499 | 0.4252 |
| HISTORY OF VALVULAR HEART DISEASE Yes | 0.5796 | 0.5103 |
| HISTORY OF GOUT Yes | -0.1974 | 0.3570 |
| HISTORY OF OBSTRUCTIVE SLEEP APNEA Yes | 0.1051 | 0.7326 |
| ICD-10 CHAPTER III Yes | 2.3522 | 0.8605 |
| ICD-10 CHAPTER IV Yes | 0.8487 | 1.1155 |
| ICD-10 CHAPTER VI Yes | 0.5940 | 1.0899 |
| ICD-10 CHAPTER VII Yes | 1.4945 | 0.6307 |
| ICD-10 CHAPTER IX Yes | 0.6583 | 0.7122 |
| ICD-10 CHAPTER X Yes | 0.6910 | 1.0891 |
| ICD-10 CHAPTER XI Yes | 1.2398 | 0.5043 |
| ICD-10 CHAPTER XIII Yes | 0.6215 | 0.6429 |
| ICD-10 CHAPTER XV Yes | 2.9987 | 1.2917 |
| ICD-10 CHAPTER XVII Yes | 2.9219 | 1.2491 |
| ICD-10 CHAPTER XVIII Yes | 0.2154 | 0.6161 |
| ICD-10 CHAPTER XIX Yes | 0.8742 | 0.8319 |
| ICD-10 CHAPTER XXI Yes | -0.1506 | 1.1094 |
| USE OF PREVENTIVE RESOURCE No | -0.0981 | 0.1815 |
| NUMBER OF HOSPITALIZATIONS STAY IN THE YEAR PRIOR TO BASELINE | -0.7197 | 0.3466 |
| LIPID LOWERING THERAPIES Yes | 0.3697 | 0.1726 |
| GLUCOSE LOWERING THERAPIES Yes | 0.0499 | 0.3297 |
| INSULIN THERAPIES Yes | -0.3294 | 0.4983 |
| ANTIPLATELETS THERAPIES AND ANTITHROMBOTIC DRUGS Yes | -0.0198 | 0.2178 |
| NITRATE THERAPIES Yes | 0.1419 | 0.6515 |
| NONSTEROIDAL ANTI-INFLAMMATORY Yes | 0.1469 | 0.2208 |
| TOTAL NUMBER OF DRUGS PRESCRIBED IN THE 3 MONTHS BEFORE BASELINE | -0.0215 | 0.0242 |
| NUMBER OF PREVIOUS ANTIYPERTENSIVE TREATMENTS IN THE YEAR BEFORE BASELINE | 0.2452 | 0.5675 |
| USE OF THIAZIDIC DIURETIC Yes | -0.7519 | 0.3801 |
| USE OF ACEI (PERINDOPRIL EXCLUDED) Yes | -0.1064 | 0.7194 |
| USE OF ARB Yes | 0.2391 | 0.6436 |
| NUMBER OF PREVIOUS ANTIHYPERTENSIVE TREATMENTS CLASSES IN THE YEAR BEFORE BASELINE | 0.0871 | 0.6253 |
| TIME SINCE FIRST ANTI-HYPERTENSIVE DRUG PRESCRIPTION | -0.0171 | 0.0227 |
| TIME FROM START OF PERINDOPRIL 4 MG OR 5MG TO BASELINE | -0.00613 | 0.00429 |
| ADHERENCE TO PERINDOPRIL TREATMENT IN THE YEAR BEFORE BASELINE | 0.0217 | 0.00750 |
| NUMBER OF BP RECORDS /PATIENT /YEAR FOR PRACTICES | -0.00668 | 0.0887 |

ACEI = angiotensin-converting enzyme inhibitor; ARB = angiotensin 2 receptor blocker; BMI = body mass index; BP = blood pressure; DBP = diastolic blood pressure; ICD-10 = *International Classification of Diseases*, 10th Revision; SBP = systolic blood pressure.

##

## Definition of Logit-PS–Matched Cohorts

PS matching was used to estimate the average treatment effect in those subjects who ultimately received treatment. In a randomized controlled trial, characteristics of the treated population will not differ systematically from the overall population (Austin, 2011). But in observational studies, and in this study specifically, the treated population differs from the overall population that is composed mostly of “control” patients. The objective of this study was to evaluate the treatment effect in the population receiving indapamide prolonged-release on top of perindopril. Patients receiving free combination were logit-PS matched to those treated with monotherapy. The logit-PS was used (instead of the PS) to obtain better-quality matching (Austin, 2011). Matching without replacement was used, with a greedy nearest neighbor within-caliper approach and a ratio of 1:3 (match of 1 patient from the free-combination arm to 3 patients in the monotherapy arm, with similar PS) as a tradeoff between bias and variance. A width of 0.2 of the standard deviation of the logit of the PS was used as a caliper (Austin, 2009).

## Evaluation of Matching Quality

Distribution overlap of PSs and logit of PSs in both treatment groups were checked before and after matching (Figure B1). A high degree of overlap in the propensity score between treated and untreated subjects after matching indicated that the means of the covariates included in the propensity score model were similar between treatment groups.

The absolute standardized difference (ASD) was used and was presented to assess covariate balance between treatment groups before and after matching (Franklin et al., 2014). The ASD was considered imbalanced if ≥0.1 (Austin, 2009). Due to the high number of covariates and the low number of patients in modalities of some covariates, some covariates remained imbalanced (ASD ≥0.1) after PS matching, including age (55-to-65-year and 65-to-75-year categories), ethnicity (Black/African/Caribbean/Black British category), BMI (underweight category), severity of hypertension (SBP ≥180 mmHg and/or DBP ≥120 mmHg category), number of BP records per patient per practice (categories of 1.5-2.5 records, 3.5-4.5 records, and ≥4.5 records), adherence to perindopril in the year before baseline, history of myocardial infarction, total number of drugs in the 3 months before baseline (class 1), and ICD-10 chapters recorded during hospitalization stays (chapter 2, “Neoplasm,” and chapter 14, “Diseases of the genitourinary system”) (see eFigure D1, eAppendix D). Although ASDs for these covariates exceeded 0.1, all were close to 0.1 after matching, and these covariates were added in the final treatment effect estimation model.

All covariates that remained imbalanced after PS matching were included in the final treatment effect model to consider residual confounding. (For variables that can be expressed both quantitatively and qualitatively and for which the quantitative form was used in the PS model, if the quantitative form remained imbalanced after matching, the qualitative form was also included in the final model.) Baseline characteristics of patients in the 2 study groups were described before and after matching.

eFigure B1. Logit-PS Density Before and After Matching


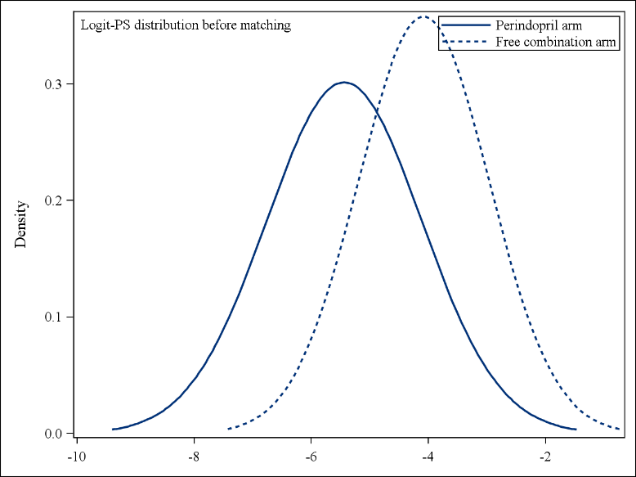

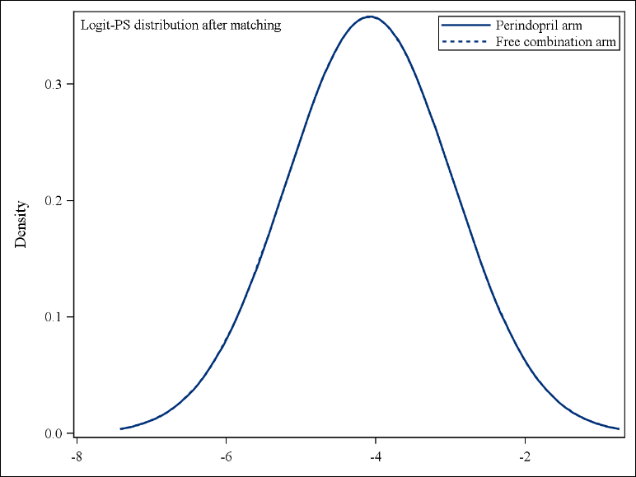


## References

Austin PC. Balance diagnostics for comparing the distribution of baseline covariates between treatment groups in propensity-score matched samples. Stat Med. 2009 Nov 10;28(25):3083-107. doi: 10.1002/sim.3697.

Austin PC. An Introduction to Propensity Score Methods for Reducing the Effects of Confounding in Observational Studies. Multivariate Behav Res. 2011 May;46(3):399-424. doi: 10.1080/00273171.2011.568786.

Franklin JM, Rassen JA, Ackermann D, Bartels DB, Schneeweiss S. Metrics for covariate balance in cohort studies of causal effects. Stat Med. 2014 May 10;33(10):1685-99. doi: 10.1002/sim.6058. Epub 2013 Dec 9.

# eAppendix C: Secondary and Sensitivity Analyses

## Secondary Analyses

A secondary methodological objective of this study was to assess different definitions of the study population and different analytical approaches to the study of BP in CPRD Aurum.

- A complete-case analysis was conducted to assess the impact of missing data. In this analysis, only patients with an SBP outcome while treated with the drug assigned at baseline (i.e., completers) were included and PS matched; all patients with missing data and/or ICEs preventing outcome measurement were excluded.
- An analysis using standardized mortality ratio (SMR) weighting (Sato and Matsuyama, 2003) instead of PS matching was conducted to make more efficient use of available data by not discarding eligible individuals who were not matched. SMR estimates the same effect as PS matching (i.e., ATT). Additionally, SMR weighting was combined with inverse probability censoring weighting (IPCW) as an alternative method to handle missing outcomes and ICEs preventing outcome measurement. IPCW weights completers to account for similar individuals without outcome records or with ICEs who are excluded from the analysis. The distribution of the weights was checked, and no truncation was needed. Variables used for IPCW models were the same as those used for the PS.
- Additional study populations were explored:
  - - - A “broader population,” comprising patients with more severe hypertension, was defined. This population included patients with uncontrolled BP at baseline, with no restriction for those who had all their SBP values ≥160 mmHg or DBP values ≥100 mmHg at the closest recorded date to baseline within the 2 weeks before baseline and baseline (included). All the other eligibility criteria were the same. In a randomized controlled trial, individuals with SBP ≥160 mmHg and DBP ≥100 mmHg would be excluded for ethical reasons, to avoid enrolling patients who could benefit from a more aggressive therapy, particularly if they were randomized to the arm with no addition of indapamide prolonged release. In this retrospective, observational study, such a situation could be non-rarely detected with no additional harm for the patient.
      - A “restricted uncontrolled population,” comprising patients with 2 successive uncontrolled SBP measurements, was included. For this population, which included patients with 2 uncontrolled BP values—defined as 2 successive SBP values ≥145 mmHg recorded on distinct dates closest to baseline, with no SBP values <145 mmHg reported on these 2 same dates—was defined. The second uncontrolled BP value had to be recorded the day of baseline or during the 2 weeks before baseline. The previous uncontrolled BP value could be reported up to 6 months before the second uncontrolled BP value, provided that no other controlled values <145 mmHg were reported in between. Those patients had to have received perindopril 5 mg or 4 mg and should not have had any other antihypertensive treatment both from the first uncontrolled BP to baseline and within 4 weeks before baseline. All the other eligibility criteria of the main populations were the same. In electronic medical record databases it is unusual to find several measures taken on not-too-distant dates (Darricarrere et al., 2023). In the restricted uncontrolled population, differences in the measurement of antihypertensive effect could be assessed when uncontrolled BP is more robustly defined.

## Sensitivity Analyses

- Different methods for calculating exposure derivation were used in sensitivity analyses (in which 15 days or 60 days between prescriptions was considered continuous treatment) than in the main analysis (in which 30 days between prescriptions was considered continuous treatment).
- The free-combination arm was restricted to patients initiating indapamide on the same date their perindopril prescription was renewed, ensuring that the prescriber’s intention was effectively to prescribe both drugs in combination.
- A time-conditional PS approach was used to address the potential for bias in the comparison of new users of free-combination therapy to prevalent users of monotherapy due to differences in disease course between treatment arms. This method was initially developed in the context of the “prevalent new-user” cohort design for comparisons between a new drug and an older drug, in order to consider all patients, including the switchers from the old to the new drug (Suissa et al., 2017). The method can also be used to compare patients adding a second treatment to a first treatment with those continuing on just the first treatment (Gagne, 2017). Because time under perindopril treatment is an important factor in measuring disease course, time-based exposure sets were used to compare patients with similar previous-exposure duration on perindopril. For each individual assigned to the free-combination arm, we identified a set of potentially eligible comparators with a similar duration of prior perindopril exposure (±1 month). A time-conditional PS was estimated for all the individuals included in the exposure sets. Individuals in the free-combination arm were PS-matched to individuals in the monotherapy arm within their exposure sets (i.e., those with the same duration of perindopril exposure before cohort entry), using the same ratio and caliper as for the primary analysis.
- A sensitivity analysis using a larger time-window for SBP outcome assessment, using the closest SBP value to 8 weeks over a period of 4 weeks to 52 weeks after baseline, also was conducted.

## References

Darricarrere C, Jacquot E, Bricout S, Louis C, Bénard M, Poulter NR. Uncontrolled blood pressure and therapeutic inertia in treated hypertensive patients: A retrospective cohort study using a UK general practice database. J Clin Hypertens (Greenwich). 2023 Oct;25(10):895-904. doi: 10.1111/jch.14699.

Gagne JJ. New-user designs with conditional propensity scores: a unified complement to the traditional active comparator new-user approach. Pharmacoepidemiol Drug Saf 2017 Apr; 26(4):469-471. doi: 10.1002/pds.4189

Sato T, Matsuyama Y. Marginal structural models as a tool for standardization. Epidemiology 2003 Nov; 14(6):680-686. doi: 10.1097/01.EDE.0000081989.82616.7d.

Suissa S, Moodie EE, Dell'Aniello S. Prevalent new-user cohort designs for comparative drug effect studies by time-conditional propensity scores. Pharmacoepidemiol Drug Saf 2017 Apr; 26(4):459-468. doi: 10.1002/pds.4107.

# eAppendix D: Population Characteristics

eTable D1. Correspondence Between Patients and Individuals^a^ in the Matched Cohort, by Treatment Group (Monotherapy Arm and Free-Combination Arm)

|  | Number of inclusions for free-combination arm | |
| --- | --- | --- |
| Number of inclusions for monotherapy arm | 0 | 1 |
| 0 | 0 | 192 |
| 1 | 548 | 1 |
| 2 | 15 | 0 |
|  | 756 patients analyzed as 772 individuals* | |

PS = propensity score.

^a^ A single patient can contribute several individuals to the study cohort at different time points.

eTable D2. Baseline Characteristics, Main Study Population, Before and After Matching

|  | Before matching | | | After matching | | |  |
| --- | --- | --- | --- | --- | --- | --- | --- |
|  | **Monotherapy (N = 22,065)** | **Free combination (N = 193)** | **ASD** | **Monotherapy (N = 579)** | **Free combination  (N = 193)** | **ASD** | **VR** |
| **Gender, n (%)** |  |  |  |  |  |  |  |
| Male | 11,982 (54.3) | 103 (53.4) |  | 294 (50.8) | 103 (53.4) |  |  |
| Female | 10,083 (45.7) | 90 (46.6) | 0.019 | 285 (49.2) | 90 (46.6) | 0.052 | 1.00 |
| **Age (years), mean ± SD** | 64.6 ± 12.4 | 61.5 ± 12.8 | 0.245 | 61.2 ± 12.1 | 61.5 ± 12.8 | 0.025 | 1.13 |
| **Age (years) in classes, n (%)** |  |  |  |  |  |  |  |
| <45 years | 1,237 (5.6) | 19 (9.8) | 0.159 | 51 (8.8) | 19 (9.8) | 0.036 | 1.11 |
| 45-55 years | 3,635 (16.5) | 41 (21.2) | 0.122 | 124 (21.4) | 41 (21.2) | 0.004 | 1.00 |
| 55-65 years | 5,930 (26.9) | 46 (23.8) | 0.070 | 181 (31.3) | 46 (23.8) | **0.167** | 0.85 |
| 65-75 years | 6,253 (28.3) | 57 (29.5) | 0.026 | 132 (22.8) | 57 (29.5) | **0.154** | 1.19 |
| 75-85 years | 3,925 (17.8) | 23 (11.9) | 0.166 | 75 (13.0) | 23 (11.9) | 0.031 | 0.93 |
| ≥85 years | 1,085 (4.9) | 7 (3.6) | 0.064 | 16 (2.8) | 7 (3.6) | 0.049 | 1.31 |
| **Ethnicity, n (%)** |  |  |  |  |  |  |  |
| White | 20,320 (92.1) | 178 (92.2) | 0.005 | 532 (91.9) | 178 (92.2) | 0.013 | 0.96 |
| Mixed/multiple ethnic groups | 37 (0.2) | - | 0.058 | <5^a^ | - | 0.083 | 0 |
| Asian or Asian British | 684 (3.1) | 6 (3.1) | 0.001 | 10 (1.7) | 6 (3.1) | 0.090 | 1.78 |
| Black/African/Caribbean/Black British | 219 (1.0) | <5^a^ | 0.055 | 9 (1.6) | <5^a^ | **0.102** | 0.34 |
| Other | 73 (0.3) | <5^a^ | 0.029 | <5^a^ | <5^a^ | 0.059 | 3.00 |
| Missing | 732 (3.3) | 7 (3.6) | 0.017 | 25 (4.3) | 7 (3.6) | 0.035 | 0.85 |
| **Socioeconomic status, n (%)** |  |  |  |  |  |  |  |
| Q1 (least deprived) | 5,211 (23.6) | 53 (27.5) | 0.088 | 157 (27.1) | 53 (27.5) | 0.008 | 1.01 |
| Q2 | 5,025 (22.8) | 57 (29.5) | 0.154 | 185 (32.0) | 57 (29.5) | 0.052 | 0.96 |
| Q3 | 4,816 (21.8) | 31 (16.1) | 0.147 | 91 (15.7) | 31 (16.1) | 0.009 | 1.02 |
| Q4 | 3,789 (17.2) | 39 (20.2) | 0.078 | 110 (19.0) | 39 (20.2) | 0.030 | 1.95 |
| Q5 (most deprived) | 3,203 (14.5) | 12 (6.2) | 0.275 | 35 (6.0) | 12 (6.2) | 0.007 | 1.03 |
| Missing | 21 (0.1) | <5^a^ | 0.077 | <5^a^ | <5^a^ | 0.059 | 3.00 |
| **Body mass index, class, n (%)** |  |  |  |  |  |  |  |
| Underweight | 182 (0.8) | <5^a^ | 0.038 | - | <5^a^ | **0.102** | NE |
| Normal | 5,128 (23.2) | 39 (20.2) | 0.074 | 119 (20.6) | 39 (20.2) | 0.009 | 0.99 |
| Overweight | 6,630 (30.0) | 72 (37.3) | 0.154 | 210 (36.3) | 72 (37.3) | 0.021 | 1.02 |
| Obesity | 4,176 (18.9) | 25 (13.0) | 0.164 | 74 (12.8) | 25 (13.0) | 0.005 | 1.02 |
| Severely obese | 2,264 (10.3) | 21 (10.9) | 0.020 | 69 (11.9) | 21 (10.9) | 0.033 | 0.93 |
| Missing | 3,685 (16.7) | 35 (18.1) | 0.038 | 107 (18.5) | 35 (18.1) | 0.009 | 0.99 |
| **Body mass index (kg/m²), mean** | 29.0 ± 5.8 | 28.9 ± 5.2 | 0.010 | 29.1 ± 5.6 | 28.9 ± 5.2 | 0.034 | 0.86 |
| **Smoking status, n (%)** |  |  |  |  |  |  |  |
| Never smoker | 5,372 (24.3) | 45 (23.3) | 0.024 | 125 (21.6) | 45 (23.3) | 0.041 | 1.06 |
| Former smoker | 12,933 (58.6) | 113 (58.5) | 0.001 | 340 (58.7) | 113 (58.5) | 0.004 | 1.01 |
| Current smoker | 3,316 (15.0) | 31 (16.1) | 0.029 | 101 (17.4) | 31 (16.1) | 0.037 | 0.94 |
| Missing | 444 (2.0) | <5^a^ | 0.004 | 13 (2.2) | <5^a^ | 0.012 | 0.93 |
| **Alcohol consumption, n (%)** |  |  |  |  |  |  |  |
| Never drinker | 2,855 (12.9) | 28 (14.5) | 0.046 | 87 (15.0) | 28 (14.5) | 0.015 | 0.98 |
| Former drinker | 2,526 (11.4) | 20 (10.4) | 0.035 | 58 (10.0) | 20 (10.4) | 0.011 | 1.03 |
| Current drinker | 12,049 (54.6) | 101 (52.3) | 0.046 | 308 (53.2) | 101 (52.3) | 0.017 | 1.01 |
| Current heavy drinker | 2,520 (11.4) | 23 (11.9) | 0.015 | 65 (11.2) | 23 (11.9) | 0.022 | 1.06 |
| Missing | 2,115 (9.6) | 21 (10.9) | 0.043 | 61 (10.5) | 21 (10.9) | 0.011 | 1.03 |
| **Year of baseline, n (%)** |  |  |  |  |  |  |  |
| 2000-2005 | 4,709 (21.3) | 58 (30.1) | 0.200 | 193 (33.3) | 58 (30.1) | 0.071 | 0.95 |
| 2005-2010 | 8,865 (40.2) | 81 (42.0) | 0.036 | 225 (38.9) | 81 (42.0) | 0.063 | 1.03 |
| 2010-2015 | 5,254 (23.8) | 37 (19.2) | 0.113 | 113 (19.5) | 37 (19.2) | 0.009 | 0.99 |
| 2015-2020 | 3,237 (14.7) | 17 (8.8) | 0.183 | 48 (8.3) | 17 (8.8) | 0.019 | 1.06 |

ASD = absolute standardized difference; CPRD = Clinical Practice Research Datalink; NE = not estimable; SD = standard deviation; VR = variance ratio

^a^ Exact data not provided, as the minimum cell count must be 5 in accordance with CPRD data governance.

eTable D3. History of Hypertension, Main Study Population, Before and After Matching

|  | Before matching | | | After matching | | |  | |
| --- | --- | --- | --- | --- | --- | --- | --- | --- |
|  | **Monotherapy  (N = 22,065)** | **Free combination (N = 193)** | **ASD** | **Monotherapy  (N = 579)** | **Free combination (N = 193)** | **ASD** | | **VR** |
| **SBP at baseline (mm Hg)** |  |  |  |  |  |  | |  |
| Mean ± SD | 150.2 ± 4.1 | 152.8 ± 4.7 | 0.592 | 152.5 ± 5.3 | 152.8 ± 4.7 | 0.053 | | 0.79 |
| Median | 150.0 | 152.0 |  | 152.0 | 152.0 |  | |  |
| Q1; Q3 | 147.0; 152.0 | 150.0; 156.0 |  | 149.0; 156.0 | 150.0; 156.0 |  | |  |
| **DBP at baseline (mm Hg)** |  |  |  |  |  |  | |  |
| Mean ± SD | 83.6 ± 8.3 | 86.1 ± 9.2 | 0.277 | 86.2 ± 8.7 | 86.1 ± 9.2 | 0.010 | | 1.13 |
| Median | 84.0 | 88.0 |  | 88.0 | 88.0 |  | |  |
| Q1; Q3 | 80.0; 90.0 | 80.0; 92.0 |  | 80.0; 92.0 | 80.0; 92.0 |  | |  |
| **Severity of hypertension, n (%)** |  |  |  |  |  |  | |  |
| SBP 145-159 mmHg and/or DBP 90-99 mmHg | 21,876 (99.1) | 186 (96.4) | 0.188 | 558 (96.4) | 186 (96.4) | 0.000 | | 1.00 |
| SBP 160-179 mmHg and/or DBP 100-119 mmHg^a^ | 185 (0.8) | 7 (3.6) | 0.190 | 18 (3.1) | 7 (3.6) | 0.029 | | 1.17 |
| SBP ≥ 180 mmHg and/or DBP ≥ 120 mmHg | <5^b^ | - | 0.019 | <5^b^ | - | **0.102** | | 0 |
| **Isolated systolic hypertension,^c^ n (%)** | 15,765 (71.4) | 101 (52.3) | 0.401 | 311 (53.7) | 101 (52.3) | 0.028 | | 1.01 |
| **Number of BP records per patient in the year before baseline** |  |  |  |  |  |  | |  |
| Mean ± SD | 4.3 ± 2.7 | 5.5 ± 3.1 | 0.401 | 5.6 ± 3.2 | 5.5 ± 3.1 | 0.027 | | 0.93 |
| Median | 4.0 | 5.0 |  | 5.0 | 5.0 |  | |  |
| Q1; Q3 | 2.0; 6.0 | 3.0; 7.0 |  | 3.0; 7.0 | 3.0; 7.0 |  | |  |
| **Number of BP records per patient in the year before baseline in class** |  |  |  |  |  |  | |  |
| 1 | 1,679 (7.6) | 7 (3.6) | 0.174 | 17 (2.9) | 7 (3.6) | 0.039 | | 1.23 |
| 2 | 4,275 (19.4) | 20 (10.4) | 0.255 | 58 (10.0) | 20 (10.4) | 0.011 | | 1.03 |
| 3 | 4,469 (20.3) | 29 (15.0) | 0.137 | 103 (17.8) | 29 (15.0) | 0.075 | | 0.88 |
| 4 | 3,322 (15.1) | 31 (16.1) | 0.028 | 89 (15.4) | 31 (16.1) | 0.019 | | 1.04 |
| ≥5 | 8,320 (37.7) | 106 (54.9) | 0.351 | 312 (53.9) | 106 (54.9) | 0.021 | | 1.00 |
| **Proportion of uncontrolled SBP per patient in the year before baseline (%), mean ±** **SD** | 83.6 ± 21.6 | 87.8 ± 17.1 | 0.220 | 88.5 ± 17.7 | 87.8 ± 17.1 | 0.035 | | 0.93 |
| **Proportion of uncontrolled DBP per patient in the year before baseline (%)** | 31.9 ± 33.7 | 46.6 ± 34.0 | 0.432 | 44.8 ± 34.9 | 46.6 ± 34.0 | 0.052 | | 0.95 |
| **Duration between BP measurements (months)** | 21,879 | 191 |  |  |  |  | |  |
| Mean ± SD | 4.14 ± 3.04 | 2.97 ± 1.83 | 0.469 | 2.93 ± 1.84 | 2.97 ± 1.83 | 0.019 | | 0.98 |
| Median | 3.36 | 2.54 |  | 2.53 | 2.54 |  | |  |
| **Type of diagnosis of hypertension** |  |  |  |  |  |  | |  |
| 2 uncontrolled BP values in the 6 months before baseline | 2,035 (9.2) | 17 (8.8) | 0.014 | 53 (9.2) | 17 (8.8) | 0.012 | | 0.97 |
| Diagnostic recorded | 20,030 (90.8) | 176 (91.2) |  | 526 (90.8) | 176 (91.2) |  | |  |
| **Time since first diagnosis of hypertension (years) (identified with a medcode), n** | 20,030 | 176 |  |  |  |  | |  |
| Mean ± SD | 5.23 ± 5.13 | 4.21 ± 5.41 | 0.194 | 4.23 ± 5.10 | 4.21 ± 5.41 | 0.004 | | 1.13 |
| Median | 3.62 | 1.92 |  | 2.38 | 1.92 |  | |  |
| **Time since first diagnosis of hypertension (years) (identified with 2 uncontrolled BP values)** | 2,035 | 17 |  | 53 | 17 |  | |  |
| Mean ± SD | 0.13 ± 0.14 | 0.16 ± 0.14 | 0.209 | 0.15 ± 0.15 | 0.16 ± 0.14 | 0.060 | | 1.12 |
| Median | 0.04 | 0.17 |  | 0.08 | 0.17 |  | |  |

ASD = absolute standardized difference; BP = blood pressure; CPRD = Clinical Practice Research Datalink; DBP = diastolic blood pressure; PS = propensity score; SBP = systolic blood pressure; SD = standard deviation; VR = variance ratio.

^a^ Patients were excluded if all their SBP measurements reported at baseline were ≥160 mmHg or all DBP ≥100 mmHg. If there was at least 1 SBP measurement <160 mmHg or <100 mmHg, it was included. As a result, some patients had their mean SBP value ≥160 mmHg and/or mean DBP ≥100 mmHg.

^b^ Exact data not provided, as the minimum cell count must be 5 in accordance with CPRD data governance.

^c^ Defined as SBP ≥145 mmHg and DBP < 90 mmHg.

eTable D4. Blood Pressure Records, Practice-Related Variables, Main Study Population, Before and After Matching

|  | Before matching | | | After matching | | |  | |
| --- | --- | --- | --- | --- | --- | --- | --- | --- |
|  | **Monotherapy (N = 22,065)** | **Free combination (N = 193)** | **ASD** | **Monotherapy (N = 579)** | **Free combination (N = 193)** | **ASD** | | **VR** |
| **Number of BP records/patient/year for practices 1 year before baseline** |  |  |  |  |  |  | |  |
| Mean ± SD | 4.38 ± 0.86 | 4.49 ± 0.84 | 0.130 | 4.55 ± 0.98 | 4.49 ± 0.84 | 0.065 | | 0.73 |
| Median | 4.32 | 4.38 |  | 4.44 | 4.38 |  | |  |
| **In class, n** (%) |  |  |  |  |  |  | |  |
| [0-1.5[ | 17 (0.1) | - | 0.039 | - | - |  | |  |
| [1.5-2.5[ | 259 (1.2) | - | 0.154 | <5^a^ | - | **0.102** | | 0 |
| [2.5-3.5[ | 2,557 (11.6) | 15 (7.8) | 0.129 | 46 (7.9) | 15 (7.8) | 0.006 | | 0.98 |
| [3.5-4.5[ | 10,266 (46.5) | 95 (49.2) | 0.054 | 252 (43.5) | 95 (49.2) | **0.114** | | 1.02 |
| ≥4.5 | 8,966 (40.6) | 83 (43.0) | 0.048 | 278 (48.0) | 83 (43.0) | 0.101 | | 0.99 |

ASD = absolute standardized difference; CPRD = Clinical Practice Research Datalink; BP = blood pressure; SD = standard deviation; VR = variance ratio.

^a^ Exact data not provided, as the minimum cell count must be 5 in accordance with CPRD data governance.

eTable D5. Antihypertensive Treatment Patterns, Main Study Population,
Before and After Matching

|  | Before matching | | | After matching | | |  | |
| --- | --- | --- | --- | --- | --- | --- | --- | --- |
|  | **Monotherapy (N = 22065)** | **Free combination  (N = 193)** | **ASD** | **Monotherapy (N = 579)** | **Free combination (N = 193)** | **ASD** | | **VR** |
| **Number of previous antihypertensive treatments in the year before baseline (perindopril excluded), mean ± SD** | 0.2 ± 0.5 | 0.3 ± 0.6 | 0.187 | 0.3 ± 0.6 | 0.3 ± 0.6 | 0.014 | | 0.99 |
| **In class (perindopril excluded)** |  |  |  |  |  |  | |  |
| 0 | 18,346 (83.1) | 143 (74.1) | 0.222 | 442 (76.3) | 143 (74.1) | 0.052 | | 1.14 |
| 1 | 2,885 (13.1) | 39 (20.2) | 0.192 | 99 (17.1) | 39 (20.2) | 0.080 | | 0.92 |
| 2 | 682 (3.1) | 10 (5.2) | 0.105 | 33 (5.7) | 10 (5.2) | 0.023 | | 0.75 |
| 3 | 120 (0.5) | <5^a^ | 0.004 | <5^a^ | <5^a^ | 0.022 | | 0 |
| ≥4 | 32 (0.1) | - | 0.045 | <5^a^ | - | 0.059 | | NE |
| **Use of thiazide diuretic in the year before baseline** | 1,270 (5.8) | 12 (6.2) | 0.019 | 32 (5.5) | 12 (6.2) | 0.029 | | 1.12 |
| **Use of ACEI (perindopril excluded) in the year before baseline** | 444 (2.0) | 6 (3.1) | 0.069 | 14 (2.4) | 6 (3.1) | 0.042 | | 1.28 |
| **Use of ARB in the year before baseline** | 137 (0.6) | <5^a^ | 0.090 | 9 (1.6) | <5^a^ | 0.000 | | 1.00 |
| **Number of previous antihypertensive treatment classes in the year before baseline (ACEI excluded), mean ± SD** | 0.2 ± 0.5 | 0.3 ± 0.5 | 0.175 | 0.3 ± 0.5 | 0.3 ± 0.5 | 0.006 | | 0.91 |
| 0 | 18,648 (84.5) | 148 (76.7) | 0.199 | 450 (77.7) | 148 (76.7) | 0.025 | | 1.14 |
| 1 | 2,798 (12.7) | 38 (19.7) | 0.191 | 102 (17.6) | 38 (19.7) | 0.053 | | 0.92 |
| 2 | 554 (2.5) | 6 (3.1) | 0.036 | 26 (4.5) | 6 (3.1) | 0.072 | | 0.75 |
| ≥3 | 65 (0.3) | <5^a^ | 0.038 | <5^a^ | <5^a^ | 0.059 | | 0 |
| **Time since first antihypertensive drug prescription (years), mean ± SD** | 5.7 ± 5.2 | 4.3 ± 4.8 | 0.277 | 4.2 ± 4.7 | 4.3 ± 4.8 | 0.011 | | 1.04 |
| Median | 4.1 | 2.3 |  | 2.4 | 2.3 |  | |  |
| **Time since start of perindopril 4 mg or 5 mg to baseline (months), mean ± SD** | 25.2 ± 30.1 | 15.6 ± 26.0 | 0.344 | 16.1 ± 20.9 | 15.6 ± 26.0 | 0.023 | | 1.55 |
| Median | 14.0 | 5.3 |  | 8.1 | 5.3 |  | |  |
| **Adherence to perindopril in the year before baseline^b^ (%), mean ± SD** | 87.4 ± 11.9 | 90.1 ± 11.7 | 0.232 | 90.6 ± 9.0 | 90.1 ± 11.7 | 0.048 | | 1.70 |
| **Adherence to perindopril in the year before baseline^b^** |  |  |  |  |  |  | |  |
| <80% | 4,025 (18.2) | 28 (14.5) | 0.101 | 55 (9.5) | 28 (14.5) | **0.155** | | 1.45 |
| ≥80% | 18,040 (81.8) | 165 (85.5) |  | 524 (90.5) | 165 (85.5) |  | |  |

ACEI = angiotensin-converting-enzyme inhibitor; ARB = angiotensin II receptor blocker; ASD = absolute standardized difference; CPRD = Clinical Practice Research Datalink; NE = not estimable; SD = standard deviation; VR = variance ratio.

^a^ Exact data not provided, as the minimum cell count must be 5 in accordance with CPRD data governance.

^b^ Adherence to perindopril treatment in the year before baseline was defined using the proportion of days covered. Proportion of days covered was calculated by taking the total number of covered days (i.e., days under perindopril prescription) in the period by the total number of days in the period. The start of the period was the day of the first record of perindopril prescription in the year before baseline and end of the period was the baseline date.

eTable D6. Comorbidities, Main Study Population, Before and After Matching

|  | **Before matching** | | | **After matching** | | |  |
| --- | --- | --- | --- | --- | --- | --- | --- |
|  | **Monotherapy (N = 22065)** | **Free combination (N = 193)** | **ASD** | **Monotherapy (N = 579)** | **Free combination (N = 193)** | **ASD** | **VR** |
| **Cardiovascular comorbidities, n (%)** |  |  |  |  |  |  |  |
| Stroke | 2,188 (9.9) | 26 (13.5) | 0.111 | 82 (14.2) | 26 (13.5) | 0.020 | 0.96 |
| Myocardial infarction | 877 (4.0) | <5^a^ | 0.189 | <5^a^ | <5^a^ | **0.112** | **5.97** |
| Heart failure | 421 (1.9) | <5^a^ | 0.127 | <5^a^ | <5^a^ | 0.000 | 1.00 |
| Periphery artery disease | 640 (2.9) | 5 (2.6) | 0.019 | 22 (3.8) | 5 (2.6) | 0.069 | 0.69 |
| Coronary artery disease | 1713 (7.8) | 8 (4.1) | 0.153 | 29 (5.0) | 8 (4.1) | 0.041 | 0.84 |
| Cardiac revascularization | 387 (1.8) | <5^a^ | 0.117 | <5^a^ | <5^a^ | 0.026 | 1.50 |
| Left ventricular hypertrophy | 119 (0.5) | <5^a^ | 0.056 | 8 (1.4) | <5^a^ | 0.032 | 0.76 |
| **Other comorbidities, n (%)** |  |  |  |  |  |  |  |
| No diabetes | 17,012 (77.1) | 146 (75.6) | 0.034 | 433 (74.8) | 146 (75.6) | 0.020 | 0.99 |
| Diabetes—HbA1c ≥6.5% | 3,317 (15.0) | 27 (14.0) | 0.030 | 75 (13.0) | 27 (14.0) | 0.030 | 1.07 |
| Diabetes—HbA1c <6.5% | 1,095 (5.0) | 13 (6.7) | 0.076 | 54 (9.3) | 13 (6.7) | 0.095 | 0.75 |
| Diabetes—unknown HbA1c | 641 (2.9) | 7 (3.6) | 0.041 | 17 (2.9) | 7 (3.6) | 0.039 | 1.23 |
| Dyslipidemia | 10,768 (48.8) | 113 (58.5) | 0.196 | 340 (58.7) | 113 (58.5) | 0.004 | 1.01 |
| Chronic kidney disease | 8,399 (38.1) | 61 (31.6) | 0.136 | 173 (29.9) | 61 (31.6) | 0.037 | 1.04 |
| Obstructive bronchopulmonary disorders | 1,177 (5.3) | 5 (2.6) | 0.141 | 14 (2.4) | 5 (2.6) | 0.011 | 1.07 |
| Metabolic syndrome | 14 (0.1) | - | 0.036 | <5^a^ | - | 0.059 | 0 |
| Atrial fibrillation | 856 (3.9) | 7 (3.6) | 0.013 | 26 (4.5) | 7 (3.6) | 0.044 | 0.82 |
| Valvular heart disease | 376 (1.7) | 5 (2.6) | 0.061 | 18 (3.1) | 5 (2.6) | 0.031 | 0.84 |
| Gout | 1,287 (5.8) | 9 (4.7) | 0.052 | 34 (5.9) | 9 (4.7) | 0.054 | 0.81 |
| Obstructive sleep apnea | 281 (1.3) | <5^a^ | 0.022 | 6 (1.0) | <5^a^ | 0.000 | 1.00 |

ASD = absolute standardized difference; CPRD = Clinical Practice Research Datalink; HbA1c = hemoglobin A1c; VR = variance ratio.

^a^ Exact data not provided, as the minimum cell count must be 5 in accordance with CPRD data governance.

eTable D7. Concomitant Treatment, Main Study Population, Before and After Matching

|  | Before matching | | | After matching | | |  | |
| --- | --- | --- | --- | --- | --- | --- | --- | --- |
|  | **Monotherapy (N = 22,065)** | **Free combination (N = 193)** | **ASD** | **Monotherapy (N = 579)** | **Free combination (N = 193)** | **ASD** | | **VR** |
| Lipid-lowering therapies | 9,639 (43.7) | 87 (45.1) | 0.028 | 262 (45.3) | 87 (45.1) | 0.003 | | NE |
| Glucose lowering—oral | 3,413 (15.5) | 31 (16.1) | 0.016 | 90 (15.5) | 31 (16.1) | 0.014 | | 1.03 |
| Glucose lowering—insulin | 834 (3.8) | 5 (2.6) | 0.068 | 19 (3.3) | 5 (2.6) | 0.041 | | 0.80 |
| Antiplatelets and antithrombotic drugs | 6,331 (28.7) | 50 (25.9) | 0.063 | 149 (25.7) | 50 (25.9) | 0.004 | | 1.01 |
| Nitrates | 559 (2.5) | <5^a^ | 0.069 | 10 (1.7) | <5^a^ | 0.014 | | 0.91 |
| Anti-angina (trimetazidine, ranolazine, nicorandil) | 98 (0.4) | - | 0.094 | <5^a^ | - | 0.083 | | 0 |
| Nonsteroidal anti-inflammatory | 2,863 (13.0) | 27 (14.0) | 0.030 | 78 (13.5) | 27 (14.0) | 0.015 | | 1.04 |
| **Total number of drugs in the 3 months before baseline, in class, mean ± SD** | 4.3 ± 4.4 | 3.7 ± 3.8 | 0.163 | 3.7 ± 3.6 | 3.7 ± 3.8 | 0.007 | |  |
| 0 | 3,133 (14.2) | 29 (15.0) | 0.023 | 102 (17.6) | 29 (15.0) | 0.070 | | 0.88 |
| 1 | 3,254 (14.7) | 41 (21.2) | 0.170 | 88 (15.2) | 41 (21.2) | **0.157** | | 1.30 |
| 2 | 2,903 (13.2) | 22 (11.4) | 0.054 | 83 (14.3) | 22 (11.4) | 0.088 | | 0.83 |
| 3 | 2,580 (11.7) | 22 (11.4) | 0.009 | 82 (14.2) | 22 (11.4) | 0.083 | | 0.83 |
| 4 | 2,144 (9.7) | 16 (8.3) | 0.050 | 46 (7.9) | 16 (8.3) | 0.013 | | 1.04 |
| ≥5 | 8,051 (36.5) | 63 (32.6) | 0.081 | 178 (30.7) | 63 (32.6) | 0.041 | | 1.04 |

ASD = absolute standardized difference; CPRD = Clinical Practice Research Datalink; NE = not estimable; SD = standard deviation; VR = variance ratio.

^a^ Exact data not provided, as the minimum cell count must be 5 in accordance with CPRD data governance.

eTable D8. Use of Healthcare Resources, Main Study Population, Before and After Matching

|  | Before matching | | | After matching | | |  |
| --- | --- | --- | --- | --- | --- | --- | --- |
|  | **Monotherapy (N = 22065)** | **Free combination (N = 193)** | **ASD** | **Monotherapy (N = 579)** | **Free combination (N = 193)** | **ASD** | **VR** |
| **Influenza vaccination in the 2 years prior to baseline, no** | 9,324 (42.3) | 92 (47.7) |  | 271 (46.8) | 92 (47.7) | 0.017 |  |
| Yes | 12,741 (57.7) | 101 (52.3) | 0.109 | 308 (53.2) | 101 (52.3) |  | 1.00 |
| **Number of hospital stays in the year prior to baseline, mean ± SD** | 0.3 ± 1.0 | 0.2 ± 0.6 | 0.126 | 0.3 ± 0.6 | 0.2 ± 0.6 | 0.052 | 0.93 |
| Median | 0.0 | 0.0 |  | 0.0 | 0.0 |  |  |
| **Number of hospital stays in the year prior to baseline in class** |  |  |  |  |  |  |  |
| 0 | 17,696 (80.2) | 162 (83.9) | 0.098 | 469 (81.0) | 162 (83.9) | 0.077 | 0.88 |
| 1 | 2,902 (13.2) | 22 (11.4) | 0.053 | 83 (14.3) | 22 (11.4) | 0.088 | 0.83 |
| 2 | 925 (4.2) | 7 (3.6) | 0.029 | 21 (3.6) | 7 (3.6) | 0.000 | 1.00 |
| 3 | 271 (1.2) | <5^a^ | 0.076 | <5^a^ | <5^a^ | 0.026 | 1.50 |
| 4 | 127 (0.6) | <5^a^ | 0.008 | <5^a^ | <5^a^ | 0.022 | 0.75 |
| ≥5 | 144 (0.7) | - | 0.115 | - | - |  | NE |

ASD = absolute standardized difference; CPRD = Clinical Practice Research Datalink; NE = not estimable; SD = standard deviation; VR = variance ratio.

^a^ Exact data not provided, as the minimum cell count must be 5 in accordance with CPRD data governance.

eTable D9. Timing of SBP Measurement Among Patients With an SBP Measurement While Treated With Baseline Strategy (i.e., “Completers”)

|  | Perindopril  (N = 345) | Free combination  (N = 115) | All (N = 460) |
| --- | --- | --- | --- |
| **Time from baseline to SBP at Week 8 (weeks)** |  |  |  |
| Mean ± SD | 11.3 ± 5.5 | 9.3 ± 4.7 | 10.8 ± 5.4 |
| Median | 10.0 | 8.0 | 9.3 |
| Q1; Q3 | 7.0; 15.3 | 5.4; 11.3 | 6.4; 14.0 |
| Min; max | 4; 24 | 4; 23 | 4; 24 |
| **Time from baseline to SBP at Week 8 (weeks) in class, n (%)** |  |  |  |
| [4 weeks – 8 weeks[ | 114 (33.0) | 53 (46.1) | 167 (36.3) |
| [8 weeks – 12 weeks[ | 99 (28.7) | 37 (32.2) | 136 (29.6) |
| [12 weeks – 16 weeks[ | 54 (15.7) | 11 (9.6) | 65 (14.1) |
| [16 weeks – 20 weeks[ | 39 (11.3) | 9 (7.8) | 48 (10.4) |
| [20 weeks – 24 weeks] | 39 (11.3) | 5 (4.3) | 44 (9.6) |

SBP = systolic blood pressure; SD = standard deviation

eFigure D1. Absolute Standardized Differences Before and After Matching, Main Study Population

| 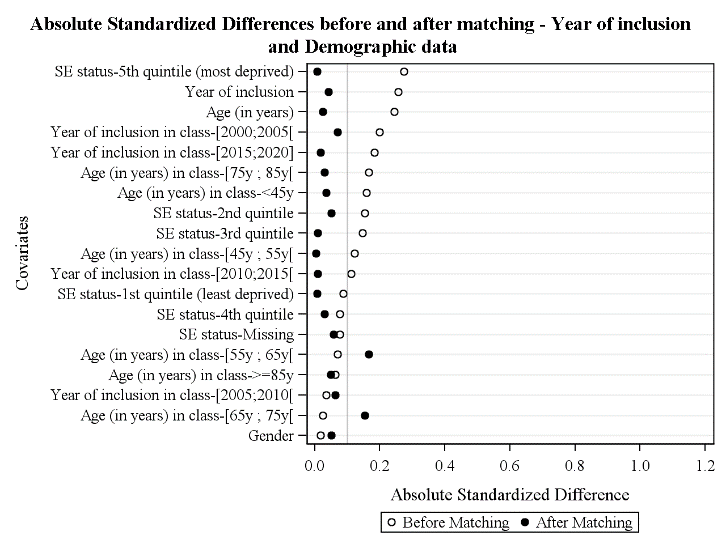 | 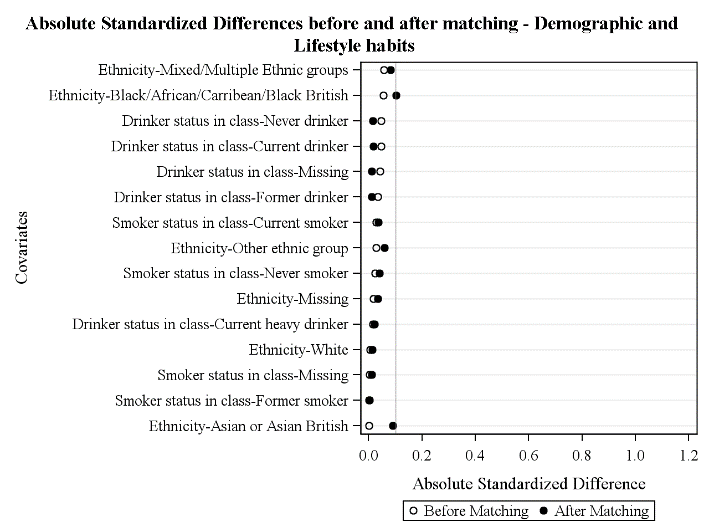 |
| --- | --- |
| 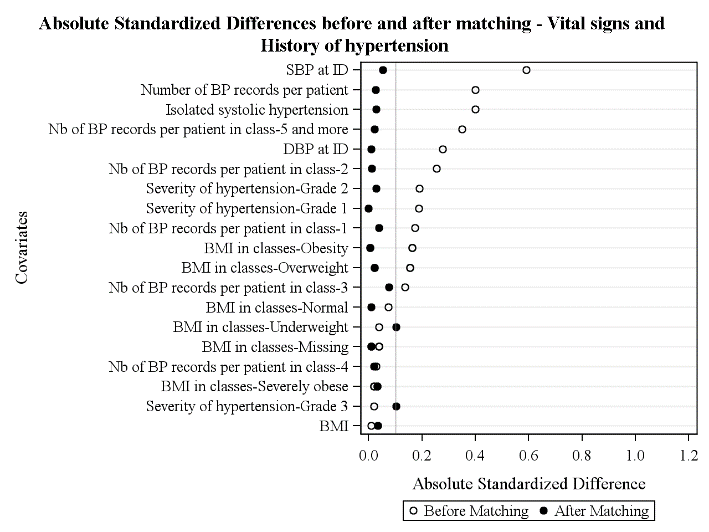 | 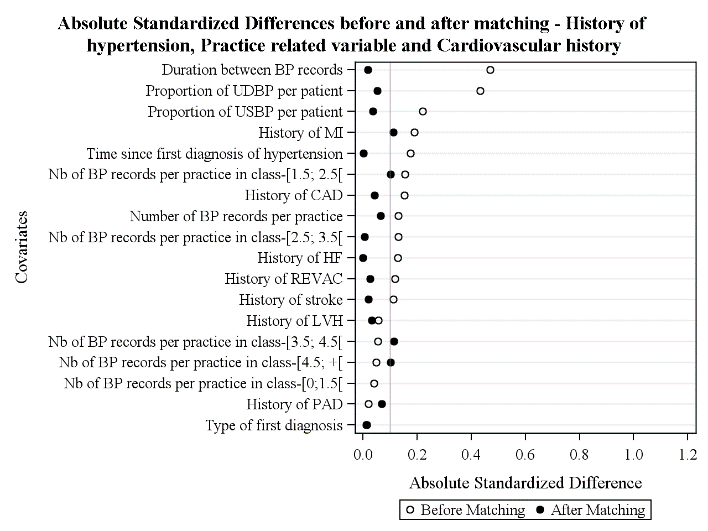 |
| 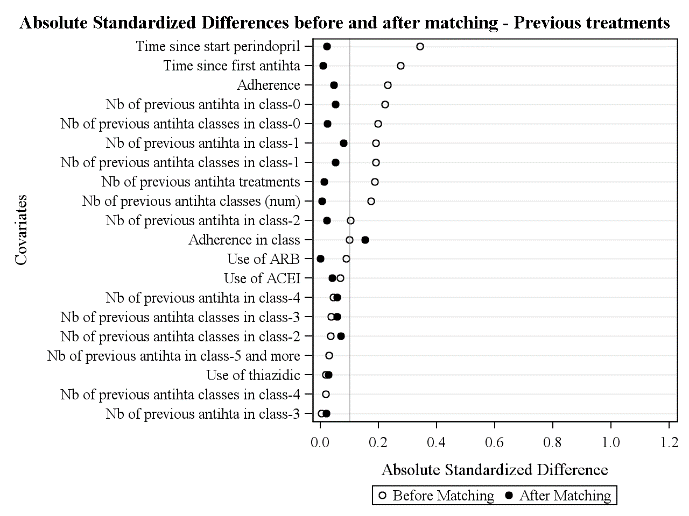 | 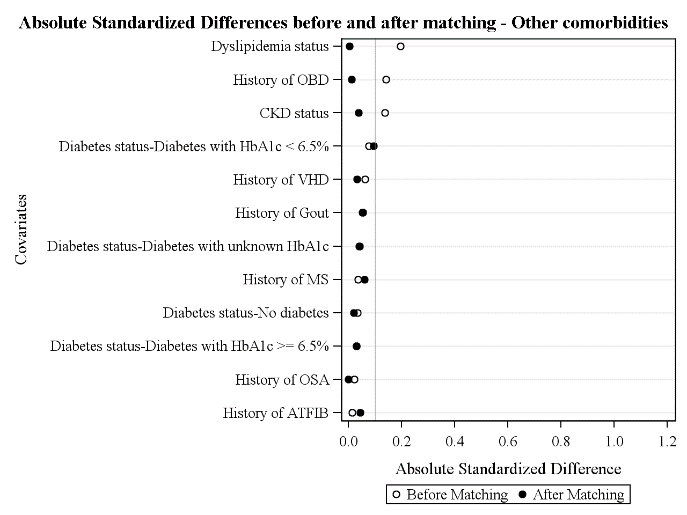 |
| 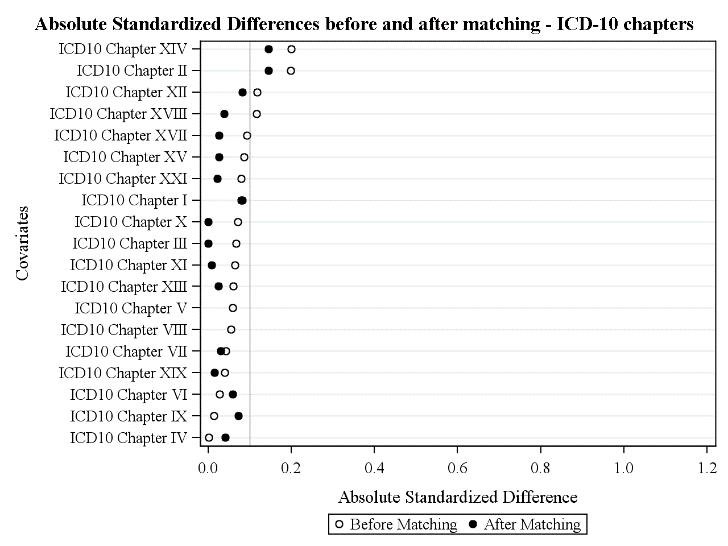 | 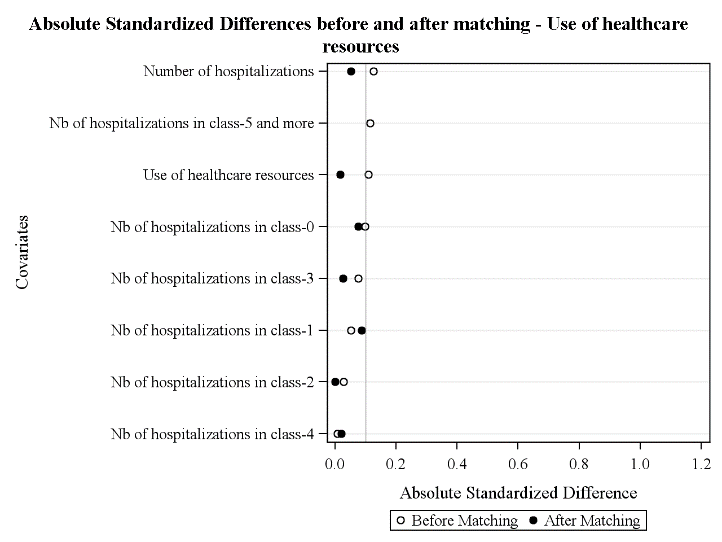 |
| 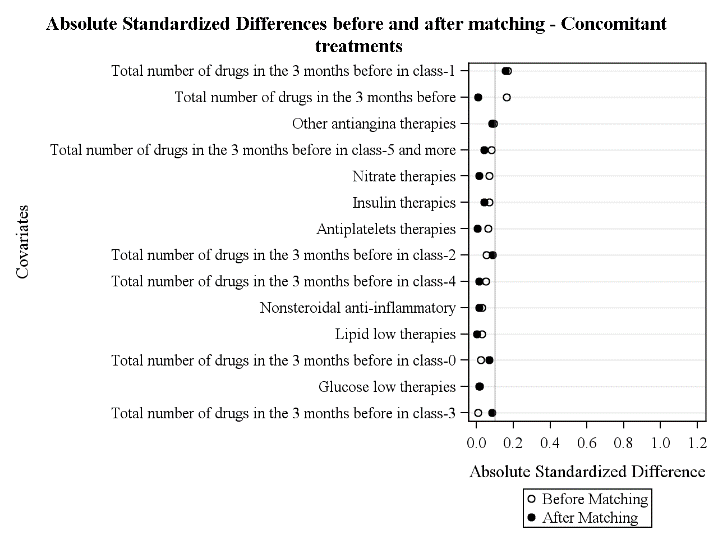 |  |

HbA1c = hemoglobin A1c; ACEI = angiotensin-converting-enzyme inhibitor; ARB = angiotensin II receptor blocker; ATFIB = atrial fibrillation; BMI = body mass index; BP = blood pressure; CAD = coronary artery disease; CKD = chronic kidney disease; DBP = diastolic blood pressure; HF = heart failure; ICD-10 = International Classification of Diseases, 10^th^ revision; ID = index date; LVH = left ventricular hypertrophy; MI = myocardial infarction; MS = metabolic syndrome; OBD = obstructive bronchopulmonary disorders; OSA = obstructive sleep apnea; PAD = periphery artery disease; SBP = systolic blood pressure; SE = socioeconomic; UDBP =uncontrolled diastolic blood pressure; USBP = uncontrolled systolic blood pressure; VHD = valvular heart disease.

Some covariates that remained imbalanced after matching included age (55-to-65-year and 65-to-75-year categories), ethnicity (Black/African/Caribbean/Black British category), BMI (underweight category), severity of hypertension (SBP ≥180 mmHg and/or DBP ≥120 mmHg category), number of BP records per patient per practice (categories of 1.5-2.5 records, 3.5-4.5 records, and ≥4.5 records), adherence to perindopril in the year before baseline, history of myocardial infarction, total number of drugs in the 3 months before baseline (class 1), and ICD-10 chapters recorded during hospital stays (chapter 2, “Neoplasm,” and chapter 14, “Diseases of the genitourinary system”). Although ASDs for these covariates exceeded 0.1, all were close to 0.1 after matching, and these covariates were added in the final treatment effect estimation model.

# eAppendix E: Additional Outcomes in Alternative Populations and With Alternative Analyses

eTable E1. Systolic Blood Pressure Change Between Baseline and Week 8: Alternative Populations

|  | **Monotherapy** | **Free combination** |
| --- | --- | --- |
| **Completers after matching (including individuals with a BP outcome while treated with the drug assigned at baseline), N = 460** | **(N = 345)** | **(N = 115)** |
| SBP at baseline (mm Hg) |  |  |
| Mean ± SD | 152.8 ± 4.6 | 152.8 ± 5.0 |
| Median | 152.0 | 152.0 |
| Q1; Q3 | 150.0; 156.0 | 150.0; 156.0 |
| Min; max | 145; 172 | 145; 170 |
| SBP at Week 8 (mm Hg) |  |  |
| Mean ± SD | 147.6 ± 14.6 | 138.2 ± 11.9 |
| Median | 146.0 | 140.0 |
| Q1; Q3 | 138.0; 156.0 | 130.0; 145.0 |
| Min; max | 107; 211 | 105; 166 |
| SBP change between baseline and Week 8 (mm Hg) |  |  |
| Mean ± SD | –5.3 ± 14.9 | –14.6 ± 12.4 |
| Median | –7.0 | –14.0 |
| Q1; Q3 | –15.0; 5.0 | –22.0; –7.0 |
| Min; max | –45; 61 | –50; 12 |
| Comparison between treatment groups of SBP change, estimate (95% CI)^a^ |  | –9.2 (–11.9 to –6.4) |
| **Broader population after matching (including uncontrolled patients with SBP ≥160 mmHg and DBP ≥100 mmHg at baseline), N = 2,332** | **(N = 1,749)** | **(N = 583)** |
| SBP at baseline (mm Hg) |  |  |
| Mean ± SD | 164.9 ± 15.0 | 165.1 ± 14.9 |
| Median | 161.0 | 160.0 |
| Q1; Q3 | 152.0; 172.0 | 154.0; 172.0 |
| Min; max | 145; 240 | 145; 236 |
| SBP at Week 8 (mm Hg) |  |  |
| Mean ± SD | 157.0 ± 18.0 | 151.8 ± 19.0 |
| Median | 155.0 | 150.0 |
| Q1; Q3 | 145.4; 167.0 | 140.0; 160.0 |
| Min; max | 105; 240 | 104; 236 |
| SBP change between baseline and Week 8 (mm Hg) |  |  |
| Mean ± SD | –7.8 ± 15.7 | –13.3 ± 16.6 |
| Median | –3.7 | –10.0 |
| Q1; Q3 | –16.0; 0.0 | –23.0; 0.0 |
| Min; max | –90; 62 | –88; 53 |
| Comparison between treatment groups of SBP change, estimate (95% CI)^a^ |  | –5.4 (–7.1 to –3.7) |
| **Patients with 2 uncontrolled BP measures after matching (N = 376)** | **(N = 282)** | **(N = 94)** |
| SBP at baseline (mm Hg) |  |  |
| Mean ± SD | 153.1 ± 4.4 | 153.4 ± 4.5 |
| Median | 153.0 | 154.0 |
| Q1; Q3 | 150.0; 157.0 | 150.0; 157.0 |
| Min; max | 145; 167 | 145; 170 |
| SBP at Week 8 (mm Hg) |  |  |
| Mean ± SD | 149.6 ± 12.8 | 143.1 ± 12.6 |
| Median | 148.7 | 144.9 |
| Q1; Q3 | 141.5; 156.0 | 137.9; 152.0 |
| Min; max | 118; 200 | 105; 172 |
| SBP change between baseline and Week 8 (mm Hg) |  |  |
| Mean ± SD | –3.5 ± 12.7 | –10.3 ± 12.6 |
| Median | –2.5 | –8.0 |
| Q1; Q3 | –9.9; 0.0 | –18.5; 0.0 |
| Min; max | –45; 50 | –50; 17 |
| Comparison between treatment groups of SBP change, estimate (95% CI)^a^ |  | –6.7 (–10.3 to –3.1) |

BP = blood pressure; CI = confidence interval; SBP = systolic blood pressure; SD = standard deviation.

^a^ Bootstrapped 2-sided 95% CI of the estimate, obtained combining estimates and standard errors using Rubin’s rule.

eTable E2. Systolic Blood Pressure Change Between Baseline and Week 8: IPCW and SMR Weightings, Completers (N = 11,427)

|  | **Monotherapy (N = 11,311)** | **Free combination (N = 116)** |
| --- | --- | --- |
| SBP at baseline (mm Hg) |  |  |
| Mean ± SD | 153.0 ± 6.4 | 152.9 ± 5.0 |
| Median | 152.0 | 152.0 |
| Min; max | 145; 188 | 145; 170 |
| SBP at Week 8 (mm Hg) |  |  |
| Mean ± SD | 146.7 ± 15.0 | 137.8 ± 11.7 |
| Median | 145.0 | 140.0 |
| Min; max | 63; 220 | 105; 166 |
| SBP change between baseline and Week 8 (mm Hg) |  |  |
| Mean ± SD | –6.3 ± 16.4 | –15.1 ± 12.5 |
| Median | –7.0 | –14.0 |
| Min; Max | –87; 75 | –50; 12 |
| Comparison between treatment groups of SBP change, estimate (95% CI)^a^ |  | –9.4 (–11.6 to –6.9) |

CI = confidence interval; IPCW = inverse probability censoring weighting; SBP = systolic blood pressure; SD = standard deviation; SMR = standardized mortality ratio.

^a^ Bootstrapped 2-sided 95% CI of the estimate, obtained combining estimates and standard errors using Rubin’s rule.

eTable E3. Systolic Blood Pressure Change Between Baseline and Week 8: Stratified analysis by calendar years and by age

|  | **Mean Difference in SBP, mmHG (95% CI)** |
| --- | --- |
| Primary analysis | -6.3 (-8.7 to -3.9) |
| Primary analysis, stratified by year |  |
| 2000-2004 | -8.4 (-12.9 to -3.9) |
| 2005-2009 | -5.1 (-8.9 to -1.3) |
| 2010-2014 | -0.7 (-6.6 to 5.28) |
| 2015-2020 | -8.2 (-19.9 to 3.4) |
| Primary analysis, stratified by age |  |
| < 55 years | -5.8 (-10.1 to -1.6) |
| 55 to 74 years | -6.0 (-9.4 to -2.7) |
| ≥ 75 years | -9.0 (-16.5 to -1.5) |

CI = confidence interval; SBP = systolic blood pressure;
